# Single-cell atlas of human liver development reveals pathways directing hepatic cell fates

**DOI:** 10.1038/s41556-022-00989-7

**Published:** 2022-09-15

**Authors:** Brandon T. Wesley, Alexander D. B. Ross, Daniele Muraro, Zhichao Miao, Sarah Saxton, Rute A. Tomaz, Carola M. Morell, Katherine Ridley, Ekaterini D. Zacharis, Sandra Petrus-Reurer, Judith Kraiczy, Krishnaa T. Mahbubani, Stephanie Brown, Jose Garcia-Bernardo, Clara Alsinet, Daniel Gaffney, Dave Horsfall, Olivia C. Tysoe, Rachel A. Botting, Emily Stephenson, Dorin-Mirel Popescu, Sonya MacParland, Gary Bader, Ian D. McGilvray, Daniel Ortmann, Fotios Sampaziotis, Kourosh Saeb-Parsy, Muzlifah Haniffa, Kelly R. Stevens, Matthias Zilbauer, Sarah A. Teichmann, Ludovic Vallier

**Affiliations:** 1 Wellcome – MRC Cambridge Stem Cell Institute, University of Cambridge, Cambridge CB2 0RE, UK; 2 Department of Surgery, University of Cambridge, Cambridge CB2 0QQ, UK; 3 Department of Paediatrics, University of Cambridge, Cambridge CB2 0QQ, UK; 4 Wellcome Sanger Institute, Hinxton CB10 1SA, UK; 5 European Molecular Biology Laboratory, European Bioinformatics Institute (EMBL-EBI), Wellcome Genome Campus, Cambridge, CB10 1SD, UK; 6 Departments of Bioengineering and Pathology, University of Washington, Seattle, Washington, USA; 7 Institute for Stem Cell and Regenerative Medicine, University of Washington, Seattle, Washington, USA; 8 NIHR Cambridge Biomedical Research Centre, Cambridge CB2 0QQ, UK; 9 Digital Institute, Newcastle University, Newcastle upon Tyne, NE4 5TG; 10 Biosciences Institute, Newcastle University, Newcastle upon Tyne, NE2 4HH, UK; 11 University of Toronto, Toronto, ON M5S 3E1, CA; 12 Multi-Organ Transplant Program, Toronto General Hospital Research Institute, Toronto, ON M5G 2C4, CA; 13 Department of Dermatology and NIHR Newcastle Biomedical Research Centre, Newcastle Hospitals NHS Foundation Trust, Newcastle upon Tyne NE2 4LP, UK; 14 Theory of Condensed Matter Group, Cavendish Laboratory, University of Cambridge, JJ Thomson Ave, Cambridge CB3 0EH, UK

## Abstract

The liver has been studied extensively due to the broad number of diseases affecting its vital functions. However, therapeutic advances have been hampered by the lack of knowledge concerning human hepatic development. Here, we addressed this limitation by describing the developmental trajectories of different cell types comprising the human liver at single-cell resolution. These transcriptomic analyses revealed that sequential cell-to-cell interactions direct functional maturation of hepatocytes, with non-parenchymal cells playing essential roles during organogenesis. We utilised this information to derive bipotential hepatoblast organoids and then exploited this model system to validate the importance of signalling pathways in hepatocyte and cholangiocyte specification. Further insights into hepatic maturation also enabled the identification of stage-specific transcription factors to improve the functionality of hepatocyte-like cells generated from human pluripotent stem cells. Thus, our study establishes a platform to investigate the basic mechanisms directing human liver development and to produce cell types for clinical applications.

The liver fulfils a broad spectrum of functions including blood detoxification, metabolite storage, lipid/glucose metabolism and secretion of serum proteins. These critical tasks are mainly performed by the hepatocytes which are supported by a diversity of cell types. Kupffer cells are tissue-specific resident macrophages responsible for liver homeostasis and immunity^1^. Hepatic stellate cells sequester vitamin A in healthy organs while promoting fibrosis through collagen secretion during disease^2^. Cholangiocytes form the epithelial lining of the biliary tree, which transports bile into the intestine^3^, and play a role in liver repair during chronic injury^4,5^. Finally, sinusoidal endothelial cells provide a permeable interface with circulating blood and promote regeneration after liver damage^6^. Importantly, adult liver cells have been broadly characterised using diverse methods, including detailed single-cell transcriptomic analyses^7–10^. However, the study of these cell types during fetal life remains limited, especially in humans due to the scarcity of descriptive studies exploring early liver development at high resolution^11^. This knowledge gap presents a major challenge in the advancement of new therapies, especially for applications of regenerative medicine. Here, we addressed this limitation by performing single-cell RNA sequencing (scRNA-seq) analyses on human fetal and adult livers (Fig. 1a). This single-cell map not only uncovered the developmental trajectories of the different cell types comprising the liver, but also the cell-to-cell interactions controlling organogenesis. We took advantage of this information to isolate human hepatoblasts, which serve as the early progenitors of the liver parenchyma, and demonstrated that they can be propagated as organoids to model developmental processes. Finally, we utilised this map to assess the differentiation path of human pluripotent stem cells (hPSCs) into hepatocyte-like cells (HLCs) and uncovered transcription factors capable of improving the resemblance of HLCs to adult hepatocytes. Together our results present insights into liver development which allow the establishment of an *in vitro* platform for modelling human liver development while providing the knowledge necessary to improve the production of hepatocytes *in vitro*^12^.

## Single-cell transcriptomic map of the developing human liver

**Fig. 1:**
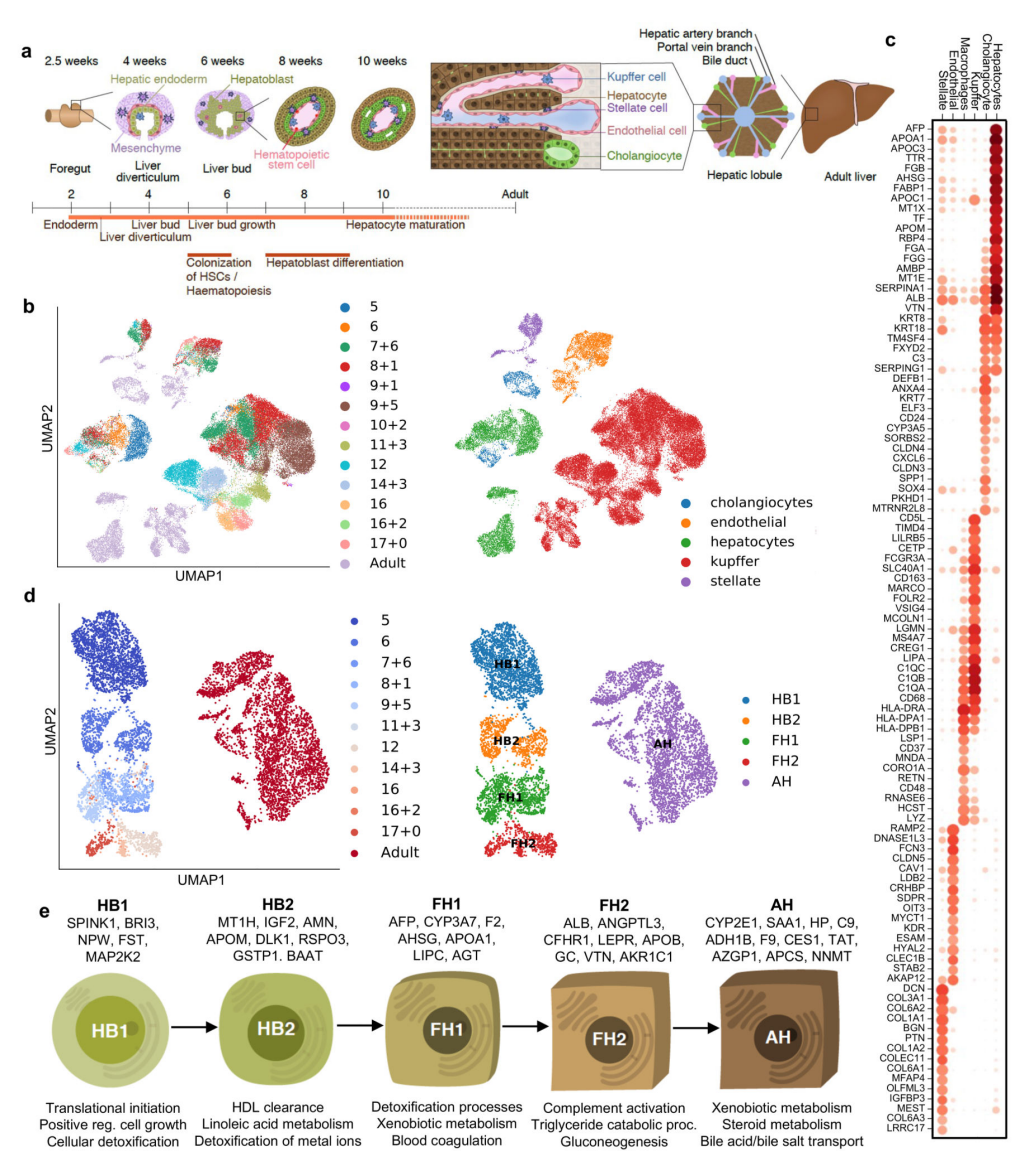
Single-cell transcriptomic map of human liver development. **a,** Schematic representation of human liver development. **b,** UMAP visualization of all integrated single-cell transcriptomic data of fetal and adult human hepatic cells generated using the 10x Genomics workflow; annotation indicates post-conceptional weeks (PCW) + days (left panel) and the cell-specific lineages (right panel). **c,** Gene expression values of selected differentially expressed genes (DEGs) for each hepatic cell lineages. Gene-expression frequency (fraction of cells within each cell type expressing the gene) is indicated by dot size and level of expression by colour intensity; colour intensity shows “gene expression [mean-scaled, log-normalized counts]”. **d,** UMAP visualization of hepatocyte developmental trajectory (left panel) and annotation of developmental stages based on Louvain analysis (right panel): HB1, hepatoblast stage 1; HB2, hepatoblast stage 2; FH1, fetal hepatocyte stage 1; FH2, fetal hepatocyte stage 2; AH, adult hepatocyte. **e,** Characteristic genes induced at each stage of hepatocyte differentiation and corresponding gene ontology (GO). Plots integrate scRNA-seq data from *n*=17 independent fetal livers aged 5-17 PCW and *n*=16 independent adult livers.

To characterise the cellular landscape of the developing liver (Fig. 1a), human single-cell transcriptomes were derived from primary tissue using methods tailored to each stage of development as well as from existing data sets (see Methods for dataset references). Droplet-based scRNA-sequencing was performed to profile a total of 237,978 hepatic cells^13^, of which 87% passed quality control^14–17^. UMAP dimensionality reduction^18^ and sub-clustering^19,20^ of these cellular transcriptomes showed that our approach captured the main cell types comprising the liver (Fig. 1b,c). Of note, cholangiocytes were the least represented cell type in our collection confirming the difficulty of isolating these cells from liver tissue^8^. In addition, cholangiocytes were only identified at 7 post-conception weeks (PCW) reinforcing previous studies indicating that these cells differentiate from hepatoblasts after 7-8 PCW^21,22^. Concerning endothelial cells, the first cells were captured from 5 PCW at the time when liver vasculature is known to be established^23,24^. Tissue-resident Kupffer cells could be distinguished from monocyte-derived macrophages by the expression of MARCO, CD163, FCGR3A and CD5L^8^ and the absence of LSP1 and CD48 (Fig. 1c). Finally, hepatic stellate cells were captured from 5 PCW, supporting studies in mice suggesting that these cells could be derived from the septum transversum at 3-5 PCW^2,25,26^. All data generated by this study is available for visualization through our online portal^27^: https://collections.cellatlas.io/liver-development. Importantly, transcriptomic observations were validated by immunostaining on primary human fetal liver (Extended Data Fig. 1a). Collectively, these results show that our single-cell atlas captured the major cell types of the liver and their dynamic diversity during development.

## Developmental trajectory of liver cells

Using this dataset, we examined the developmental trajectory of each cell type, starting with hepatocytes. Principal component analysis (PCA), Louvain clustering and diffusion pseudotime (dpt) analyses^28,29^ defined 5 hepatocyte developmental stages (Fig. 1d; Extended Data Fig. 1): hepatoblast stages 1 and 2 (HB1 at 5 PCW and HB2 at 6 PCW), fetal hepatocyte stages 1 and 2 (FH1 at 7-11 PCW and FH2 at 12-17 PCW) and adult hepatocytes (AH). Each stage displayed distinct transcriptional changes indicative of unique cell states (Extended Data Fig. 1b-e). Accordingly, this analysis identified markers specific to each stage including SPINK1 for hepatoblasts, GSTA1 for fetal hepatocytes, and haptoglobin (HP) for adult hepatocytes. We also observed that each stage of hepatocyte development was marked by the induction of genes associated with specific liver function (Fig. 1e). Thus, hepatocytes follow a progressive functional maturation during organogenesis corresponding to the acquisition of hepatic activity during fetal life.

**Fig. 2:**
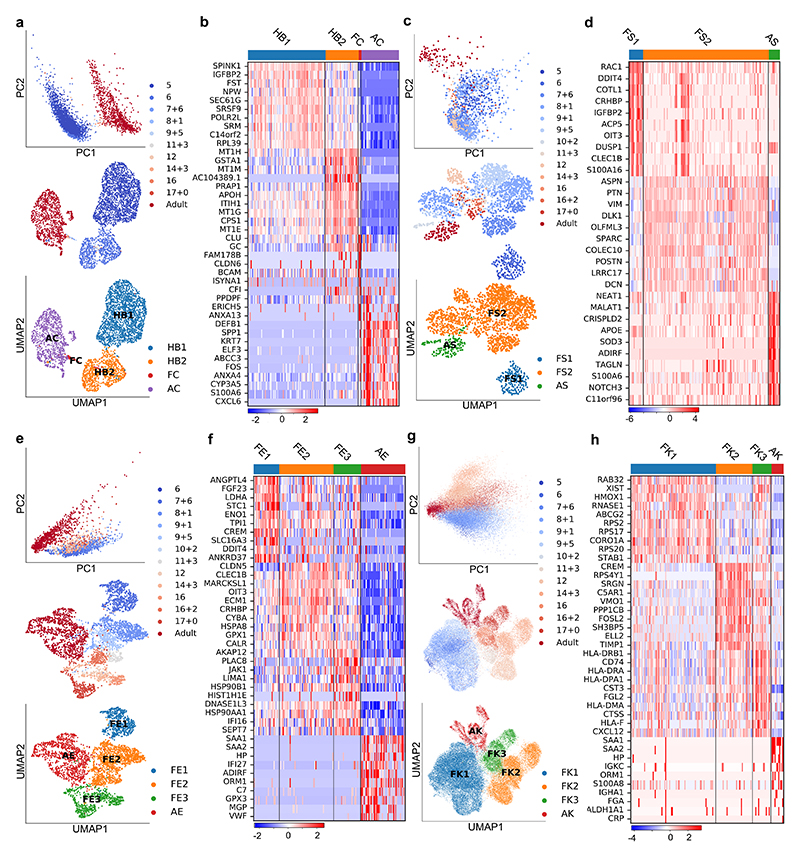
Mapping nonparenchymal cell identity during human liver development. **a,** PCA (top) and UMAP (middle) plots of primary human cholangiocyte sample timepoints and UMAP annotation of discrete cholangiocyte developmental stages (bottom); HB1 = hepatoblast 1, HB2 = hepatoblast 2, FC = fetal cholangiocyte, AC = adult cholangiocyte. **b,** Heatmap showing time-related DEGs of each stage of primary cholangiocyte development. **c,** PCA (top) and UMAP (middle) plots of primary human hepatic stellate cell sample timepoints and UMAP annotation of discrete stellate cell developmental stages (bottom); FS1 = fetal stellate cell 1, FS2 = fetal stellate cell 2, AS = adult stellate cell. These three developmental stages correlate with the onset of haematopoietic function of the liver and birth. **d,** Heatmap showing time-related DEGs of each stage of primary hepatic stellate cell development. **e,** PCA (top) and UMAP (middle) plots of primary human endothelial cell sample timepoints and UMAP annotation of discrete endothelial cell developmental stages (bottom); FE1 = fetal endothelial cell 1, FE2 = fetal endothelial cell 2, FE3 = fetal endothelial cell 3, AE = adult endothelial cell. **f,** Heatmap showing time-related DEGs of each stage of primary endothelial cell development. Endothelial cells are closely associated with haematopoietic stem cell differentiation, with changes of function associated with haematopoietic and vascularization events. **g,** PCA (top) and UMAP (middle) plots of primary human Kupffer cell sample timepoints and UMAP annotation of discrete Kupffer cell developmental stages (bottom); FK1 = fetal Kupffer cell 1, FK2 = fetal Kupffer cell 2, FK3 = fetal Kupffer cell 3, AK = adult Kupffer cell. **h,** Heatmap showing time-related DEGs specific to each stage of primary Kupffer cell development. Heatmap colour scales show “gene expression [mean-scaled, log-normalized counts]”. Plots integrate scRNA-seq data from *n*=17 independent fetal livers aged 5-17 PCW and *n*=16 independent adult livers.

We then performed similar analyses on cholangiocytes, stellate cells, endothelial cells, and Kupffer cells (Fig. 2). Briefly, only cholangiocytes seemed to gradually differentiate from the HB2 stage, whereas PCA analyses did not reveal major differences among sequential timepoints for most non-parenchymal hepatic cell types. More precisely, Louvain clustering and diffusion pseudotime allowed for the distinction of an embryonic stage at 5-6 PCW, intermediate fetal stage between 7-17 PCW, and an adult state (Fig. 2a,c,e,g). This suggested that these cell types may not undergo a significant functional maturation during fetal life after their initial embryonic specification. Interestingly, these three stages correspond to major modifications in the liver environment: liver bud formation, colonisation by the haematopoietic system at 7 PCW and the shift from fetal to adult cells^24^. Thus, these data suggest that the developmental trajectory of hepatic cells is influenced by major developmental events while only hepatocytes seem to undergo a progressive functional maturation.

**Fig. 3:**
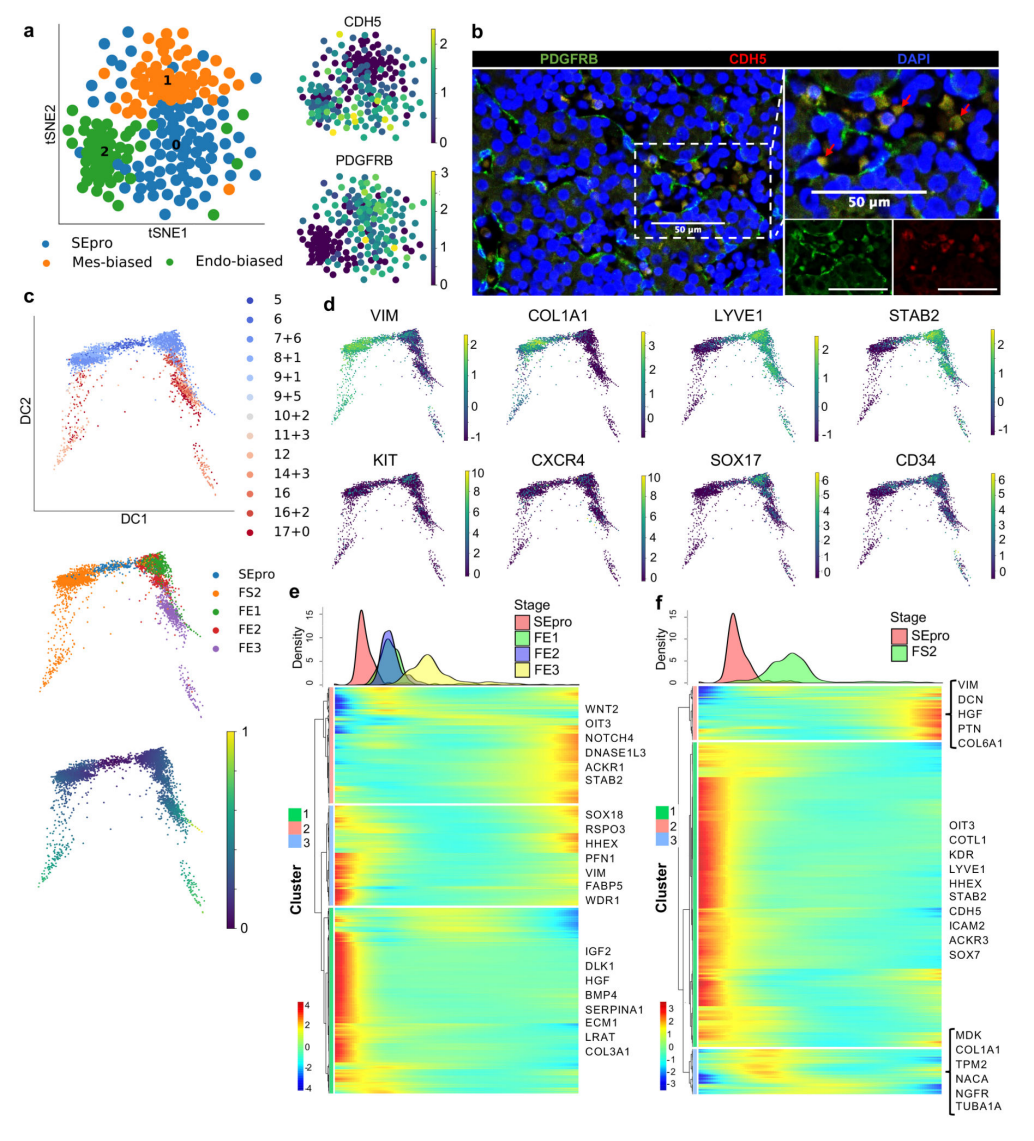
Identification of a hepatic stellate and endothelial cell progenitor in the early fetal liver. **a,** tSNE visualization based on Louvain clustering of 6 PCW human fetal liver cells identifying stellate-endothelial progenitors or “SEpro” (left panel). Gene expression tSNE plots show the co-expression of specific markers for both hepatic stellate and endothelial lineages by SEpros (right panel) (*n*=3 independent fetal livers). **b,** Immunofluorescence staining of 6 PCW human liver identifying the SEpro population based on co-expression of stellate (PDGFRB) and endothelial (CDH5) markers; scale bars = 50 um. **c,** Diffusion pseudotime analyses of stellate and endothelial cells developmental trajectories showing that each lineage originated from SEpro (integrated scRNA-seq data from *n*=17 independent fetal livers aged 5-17 PCW). **d,** Diffusion pseudotime analyses of specific markers for each lineage (top row stellate cells, bottom row endothelial cells). **e,** Heatmap of time-related genes during fetal endothelial cell development and **f,** fetal hepatic stellate cell development starting with SEpro and progressing toward 17 PCW. Dpt pseudotime colour scale shows “geodesic distance [distance between nodes]”; heatmap colour scale shows “gene expression [mean-scaled, log-normalized counts]”.

We then further demonstrated the utility of our single-cell map in defining the embryonic origin of specific cell types. We decided to focus on hepatic stellate cells, since previous studies have reached divergent conclusions^2^. Louvain clustering on early stellate cells revealed a population of cells expressing mesenchymal markers, one population with an endothelial-bias, and a third population combining markers for both lineages (endothelial: CDH5, LYVE1, KDR and STAB2 and stellate cells: PDGFRB, VIM, DES and COL1A1; Fig. 3a-c). Diffusion pseudotime confirmed that fetal endothelial and stellate cells could originate from this stellate-endothelial progenitor population, termed SEpro (Fig. 3c-f), while gene expression analyses reveal that these cells expressed genes associated with proliferation and DNA replication characteristics of a stem cell or progenitor state. Immunohistochemistry validations on primary tissue revealed cells expressing both PDGFRB and CDH5 located within the vasculature of the 6 PCW liver (Fig. 3b), thereby confirming the existence of this progenitor *in vivo*. Of note, previous studies in model organisms have suggested the existence of such progenitors without functional demonstration^30,31^. To further address this limitation, we decided to validate the existence of such progenitors during differentiation of hPSCs *in vitro*. We first performed scRNA-seq analyses on hPSCs differentiating into endothelial^32^ and hepatic stellate cells^33^. UMAP, PCA and diffusion pseudotime comparison of these differentiations reveal an overlapping stage sharing the expression of markers specific for both lineages (Extended Data Fig. 2a-d). To confirm that this stage could include a common progenitor, hPSC were differentiated into endothelial cells for 3.5 days and then grown in culture conditions inductive for hepatic stellate cells. The resulting cells were able to transition away from the endothelial pathway characterised by the expression of CDH5, KDR and VWF while acquiring the stellate cells markers PDGFRA, COL1A1, ACTA2, and NCAM (Extended Data Fig. 2e-f). Taken together, these results illustrate how single cells observations can be combined with in vitro differentiation to further understand the developmental process leading to stellate cells production.

## Human hepatoblasts organoids derivation and differentiation

**Fig. 4:**
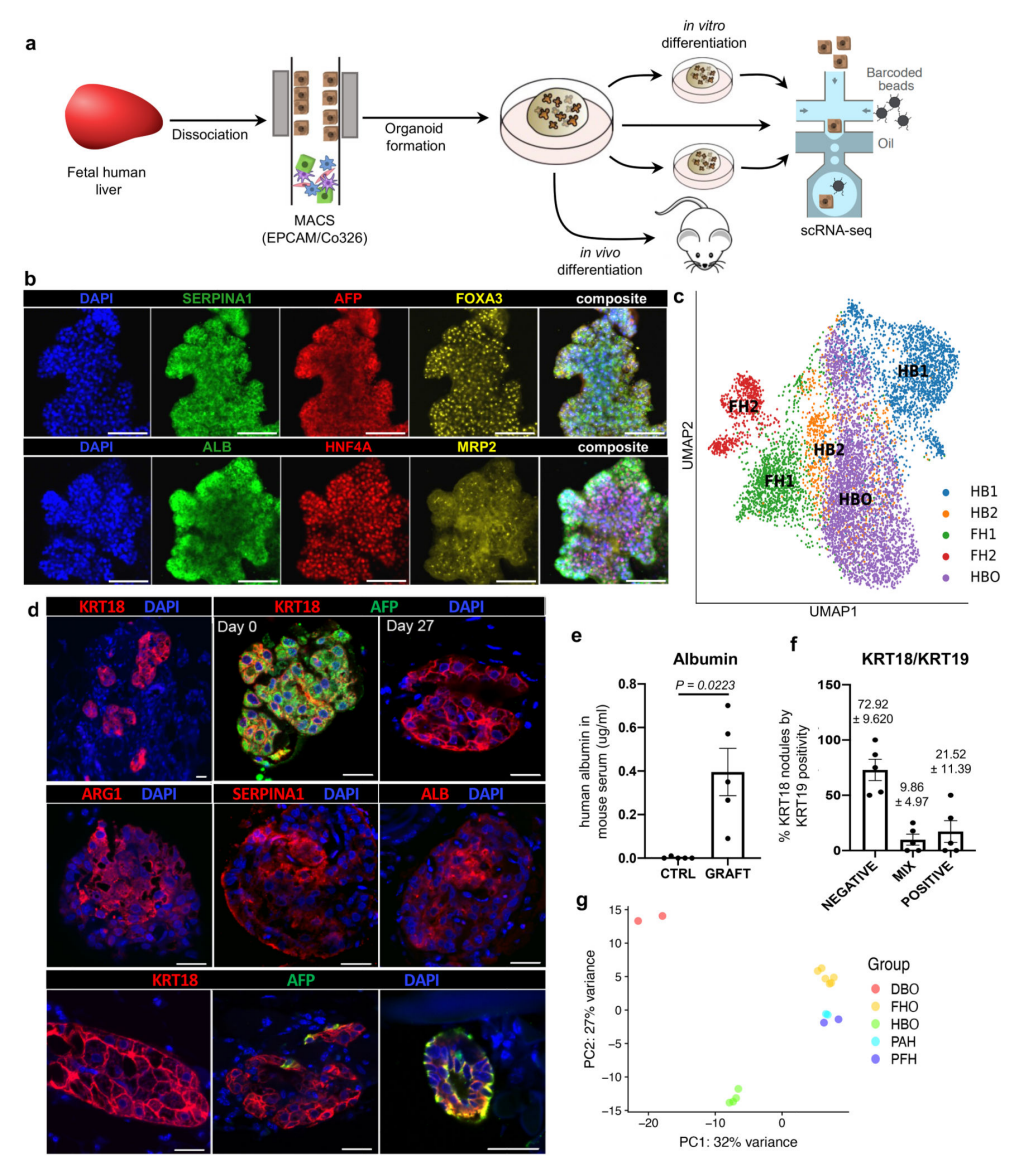
Modelling early hepatic development *in vitro* using hepatoblast organoids. **a,** Schematic representation of hepatoblast organoid (HBO) derivation and subsequent analyses. **b,** Immunostaining of hepatoblast markers in HBO grown *in vitro*; scale bars = 100 um. **c,** UMAP visualisation of fetal hepatoblast/hepatocyte differentiation stages along with HBO, confirming that HBO share the transcriptional profile of the HB2 stage of hepatocyte development. **d,** Immunostaining showing decrease of the fetal hepatocyte marker (AFP) in HBO after 27 days of engraftment while hepatocyte markers (KRT18, ALB, ARG1, and SERPINA1) were maintained, indicative of differentiation into mature hepatocytes. Immunostaining for biliary markers identified KRT19-positive cells in a subset of nodules, which organised into bile duct-like structures. Unless otherwise stated, pictures show grafts 27 days post-transplantation; scale bars = 20 um. **e,** ELISA analyses showing secretion human ALB in the serum of HBO recipient mice 27 days after engraftment *(n=5* independent animals). **f,** Quantification (percentage) of KRT19-positive cells within KRT18-positive nodules. **g,** Principal component analysis (PCA) showing the divergence in gene expression profile between hepatoblast organoids (HBO; *n=4* lines derived from 4 independent fetal livers), differentiated biliary organoids (DBO; *n=*2), fetal hepatocyte organoids (FHO; *n=*6), primary adult hepatocytes (PAH; *n=*2), and primary fetal liver (PFH; *n=*2). Data are presented as mean values +/- SEM; unpaired two-tailed t-tests.

We then decided to use our single-cell analyses to isolate and grow *in vitro* hepatoblasts, as they represent the natural stem cell of the liver during development. Our single-cell analyses showed that hepatoblast HB1 and HB2 display characteristics of early these liver stem cells. Indeed, these cells expressed WNT target genes associated with adult stem cells such as LGR5, high levels of cell cycle regulators suggesting self-renewal capacity, and markers specific for both biliary and hepatocytic lineages indicative of bi-potential capacity of differentiation (Extended Data Fig. 1e). Based on these observations and previous reports showing a crucial role for WNT^34^, we hypothesised that this signalling could support the growth of hepatoblasts *in vitro*. To explore this possibility, 6 PCW livers were dissociated into single cells which were sorted based on EPCAM expression and grown in 3D culture conditions supplemented with WNT (Fig. 4a). The isolated cells formed branching organoids which could be expanded for more than 20 passages (Extended Data Fig. 3a-b). These hepatoblast organoids (HBO) homogeneously expressed hepatoblast markers (Fig. 4b; Extended Data Fig. 3c-d) and single cell RNA-seq analyses demonstrated that they closely resembled their *in vivo* counterparts, especially the HB2 stage (Fig. 4c; Extended Data Fig. 3e-f). Of note, neither HBO nor HB1/2 cells express NCAM, thereby excluding the presence of hepatic stem cells in our analyses^35^. To confirm HBO bipotentiality, we transplanted tdTomato-HBO into Fah-/Rag2-/Il2rg- (FRG) mice^36^ using an approach developed for primary hepatocytes^37,38^. After 27 days, implants were recovered, and red fluorescent cells could be observed in all the grafts (Extended Data Fig. 3g) indicating that HBO had engrafted efficiently. H&E staining of explanted tissue sections revealed the presence of numerous nodules resembling densely packed hepatocytes, as well as biliary epithelial-like cells assembled into structures resembling bile ducts (Extended Data Fig. 3h). Engrafted organoids stained positive for both KRT18 and AFP at the time of implant, but AFP was markedly decreased by day 27 (Fig. 4d) suggesting differentiation into hepatocytes *in vivo*. Accordingly, numerous cells in hepatic nodules stained positively for ARG1, A1AT, and ALB while significant levels of human albumin were identified in mouse serum suggesting functional activity of implanted organoids (Fig. 4e). Finally, some KRT18+ nodules were found to contain cells expressing KRT19 (Fig. 4f), either as a mixed population or as a pure KRT19+ population. Together, our results demonstrate that HB2 hepatoblasts can be grown *in vitro* while maintaining their capacity to differentiate into hepatocytes and cholangiocytes.

Of note, two types of human liver organoid systems have been described previously by Huch et al. (2015)^39,40^ and Hu et al. (2018)^36^. The former is composed of intrahepatic cholangiocytes which can differentiate towards hepatocyte-like cells^39,41^ (differentiated biliary organoids or DBO), whilst the latter derives organoids from hepatocytes^36^. Therefore, we characterised both systems against HBO (Fig. 4g). Organoids derived from intrahepatic cholangiocytes expressed markers such as KRT19, but did not express hepatocyte markers in either the undifferentiated nor differentiated state (Extended Data Fig. 3i-j). Transcriptomic comparison also demonstrated the transcriptional divergence between HB2/HBO, DBO and hepatocyte organoids (Fig. 4g). Analysis of the genes driving this separation revealed hepatocyte and biliary markers, with HBO having intermediate levels of both these sets of markers (Extended Data Fig. 3i-l). Taken together, these data demonstrate that our single-cell analyses have identified a unique self-renewing population of hepatoblasts that can be propagated long-term *in vitro*.

## Dynamic intercellular interactions of the developing liver

**Fig. 5:**
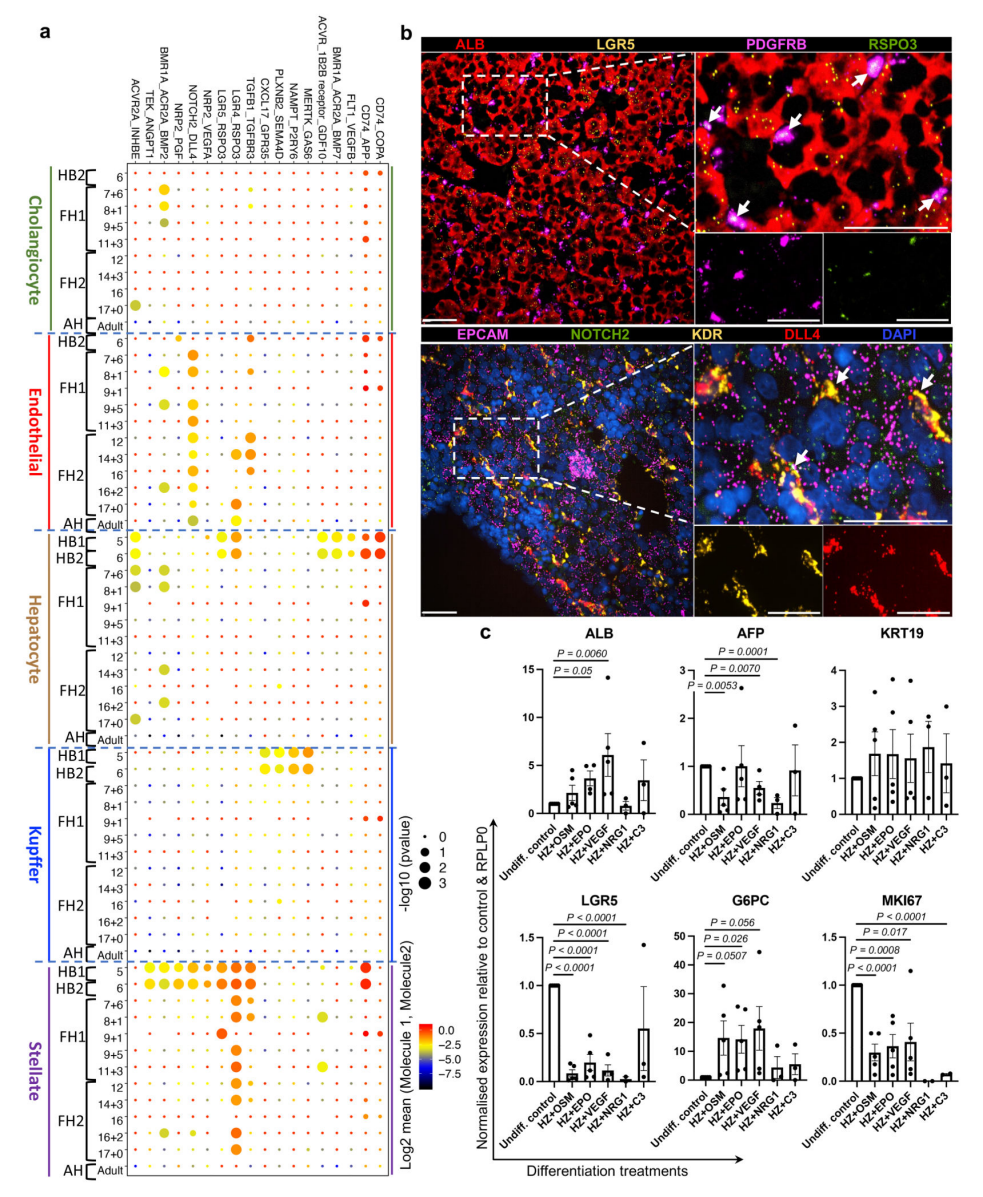
Cell-to-cell interaction networks during human liver development. **a,** CellphoneDB analysis of the receptor-ligand interactions of hepatocytes with other hepatic cells across all developmental timepoints. Y-axis shows ligand-receptor/receptor-ligand interactions, with the hepatocyte protein listed first in each pairing; x-axis shows developmental timeline of each cell type; dot colour signifies log2 mean expression of interacting molecules and dot size shows -log10(P) significance (integrated scRNA-seq data from *n*=17 independent fetal livers ranging in age from 5 to 17 post-conceptional weeks; *n*=16 independent adult livers). **b,** RNAscope validating ligand-receptor interactions that establish the hepatoblast niche in 6 PCW liver. RSPO3 is expressed in hepatic stellate cells and its LGR5 receptor is expressed by hepatoblasts (top panels). DLL4 is expressed by endothelial cells while NOTCH2 receptor is expressed on hepatoblasts (bottom panels); scale bars = 50 um. **c,** Quantitative PCR showing the expression of hepatocyte maturation genes following treatment with key signalling molecules discovered using the single-cell liver development atlas (*n=*5 independent experimental replicates); Undiff. control = HBO grown in upkeep culture conditions to maintain their self-renewal capacity, HZ = HepatoZYME basal medium. Data are presented as mean values +/- SEM; unpaired two-tailed t-tests.

To further understand the mechanisms directing liver organogenesis, we captured the interactions between the hepatoblasts/hepatocytes and other cell types using the CellPhone database (CellPhoneDB)^42,43^ (Fig. 5a; Extended Data Fig. 4). This approach revealed that most interactions begin in hepatoblasts, stabilise in fetal hepatocytes and finally disappear in adult cells (Extended Data Fig. 4a). Furthermore, a diversity of unknown interactions was captured between stellate cells, Kupffer cells and endothelial cells indicating potential roles in extracellular matrix organisation, haematopoietic development and innate immunity (Extended Data Fig. 4a). Thus, our analysis could reveal the source of signalling pathways controlling liver development. Several of these interactions were validated using RNA-Scope on primary liver tissues (Extended Fig. 4b-d). As an example, NOTCH4/DLL4 was expressed by endothelial cells and could interact with DLK1/NOTCH2 on hepatoblasts/hepatocytes (Fig. 5b and Extended Data Fig. 4c). These bidirectional interactions suggest that hepatoblasts could be involved in the vascularisation of the liver and thus could direct the construction of their own niche. In return, endothelial cells could control hepatoblast differentiation into cholangiocytes, a process known to require NOTCH signalling^44^. Similarly, RSPO3-LGR4/5 interactions were detected between hepatoblasts and stellate cells at 5-6 PCW (Fig. 5a-b). Thus, stellate cells could support hepatoblast self-renewal by boosting WNT signalling.

**Fig. 6:**
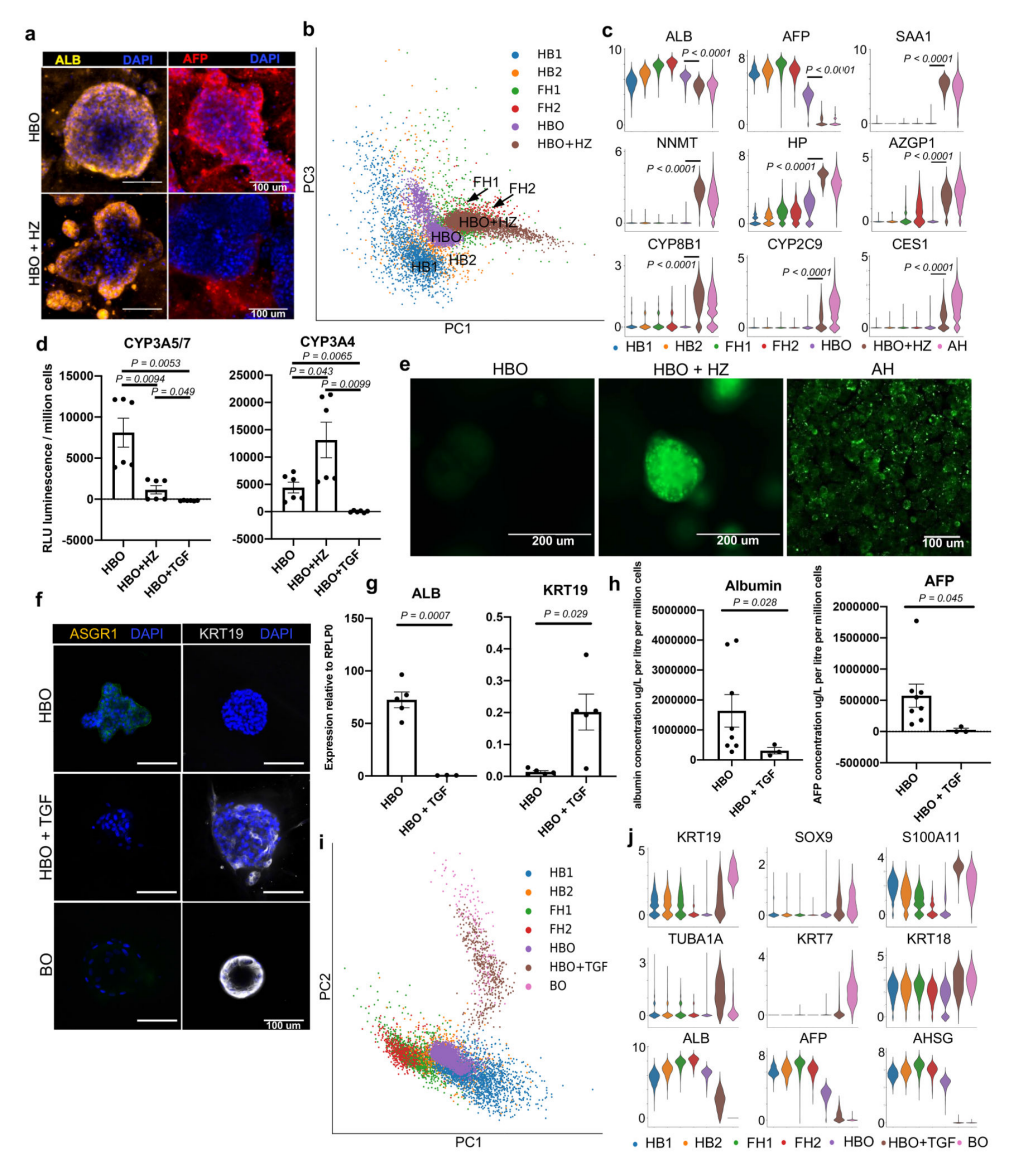
Characterisation of the differentiation capacity of hepatoblast organoids into both hepatocyte and cholangiocyte mature lineages. **a,** Immunostaining showing that HBO differentiated into hepatocytes (HBO+HZ) maintain expression of ALB while losing the fetal marker AFP; scale bars = 100 um. **b,** PCA showing that HBO differentiation *in vitro* follows the developmental trajectory of fetal primary hepatocyte development. **c,** Violin plots of key functional markers corresponding to the acquisition of an adult hepatocyte phenotype after HBO differentiation. **d,** Cytochrome P450 3A5/7 and cytochrome P450 3A4 activity in HBO, HBO+HZ, and HBO treated with TGFB (HBO+TGF) (*n*=6 independent experimental replicates using lines derived from 2 independent fetal livers). **e,** BODIPY assay showing differences in lipid uptake in HBO compared to HBO + HZ and primary adult hepatocytes (PAH). **f,** Immunocytochemistry of HBO, HBO+TGF, and BO stained for KRT19 and ASGR1; scale bars = 100 um. **g,** QPCR analyses showing the expression of denoted genes in HBO and HBO+TGF (*n*=5, each point represents an HBO line derived from a unique primary fetal liver). **h,** ELISA analyses showing the concentration of protein in media secreted by HBO (*n*=8, each line derived from an independent fetal liver), and HBO treated with TGFB (*n*=3) after 48 hours of freshly applied medium. Values are normalised to cell number (i.e. per million cells) with albumin as micrograms per litre, and alpha-fetoprotein as units per ml. **i,** PCA plot of scRNA-seq data comparing the *in vitro* differentiation of HBO toward cholangiocytes (HBO+TGF) to adult biliary organoids and *in vivo* differentiation of hepatoblasts. **j,** ScRNA-seq violin plots showing the loss of hepatocyte functional genes and the acquisition of a biliary transcriptome, thus demonstrating the similarity of HBO+TGF cholangiocytes to the positive biliary organoid control. Data are presented as mean values +/- SEM; unpaired two-tailed t-tests.

We then decided to test if these analyses could also be used to identify pathways controlling hepatocyte and cholangiocyte differentiation. For the former, we selected 5 growth factors suggested by CellphoneDB analyses (EGF, VEGFA, C3 NRG1 and EPO, Oncostatin-M or OSM) (Fig. 5a, Extended Data Fig. 4). The effect of these factors was then analysed on HBO grown in the absence of WNT to allow their differentiation. Several factors (EPO, OSM and VEGFA) were associated with a decrease in hepatoblast markers (AFP, LGR5 and MKI67) and increase in hepatocyte markers (albumin and G6PC) (Fig. 5c; Fig. 6a) suggesting a differentiation toward fetal hepatocytes. Other factors either blocked the expression of hepatocytes markers (NRG1) or have limited effect (C3). To further reinforce these observations, we decided to characterise the effect of OSM by performing scRNA-seq on HBO induced to differentiate into hepatocytes. These analyses confirmed the transcriptional shift of HBO toward hepatocyte after treatment with OSM (Fig. 6b,c). Furthermore, this differentiation was associated with gain of hepatocyte functions including cytochrome P450 activity (Fig. 6d) and lipid accumulation (Fig. 6e). Together, these data confirm that HBO can differentiate into hepatocytes in the absence of WNT upon stimulation of specific factors such as OSM.

Focusing next on cholangiocyte differentiation, CellphoneDB analyses reinforced previous reports^4,5^ suggesting an important function for TGFB in this process^45^ (Fig. 6a). To test this hypothesis, we supplemented HBO media with TGFB for seven days and observed a marked switch toward cholangiocyte identity illustrated by the induction of biliary markers KRT19 and loss of hepatoblast markers (Fig. 6d,f-h). These observations were confirmed by scRNA-seq analyses showing that the transcriptome of HBO grown in the presence of TGFB resemble that of cholangiocyte organoids (Fig. 6d,i,j) Taken together, these data confirm the interest of our single cell analyses to identify cell-cell interactions directing liver development and also the interest of HBO for validating the function of the signalling pathways involved.

## Liver atlas informs the maturation of hiPSC-derivatives

**Fig. 7:**
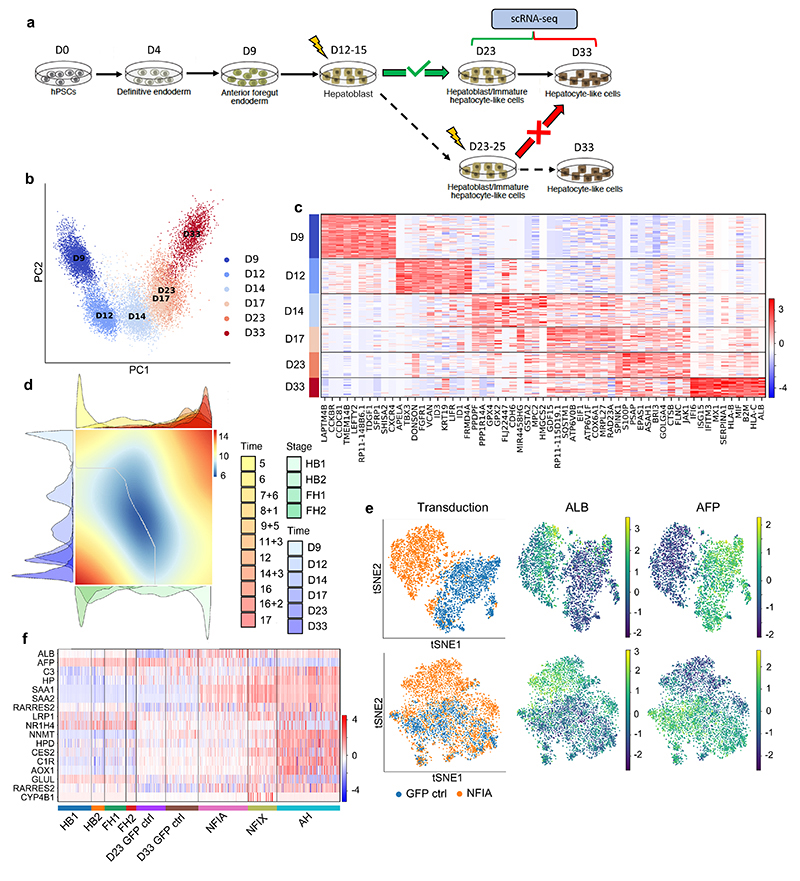
Temporal overexpression of key transcription factors in hPSC-derived hepatocytes increases their similarity to adult primary hepatocytes. **a,** Schematic representation of experimental processes to validate the functional transcription factors in hepatocyte differentiation. b, PCA showing the step-by-step differentiation of hPSCs into hepatocytes. D: day of differentiation (*n*=6 sequential differentiation timepoints, with one replicate sequenced per timepoint). c, Heatmap of top 10 differentially expressed genes (DEG) specific between each stage of differentiation; Wilcoxon-Rank-Sum test, z-score>10. d, Alignment of primary hepatocyte developmental trajectory to hiPSC differentiation using the CellAlign software; red colour shows regions of misalignment/dissimilarity, blue colour shows regions of close alignment/similarity (integrated scRNA-seq data from *n*=17 independent fetal livers ranging in age from 5 to 17 post-conceptional weeks and *n*=16 independent adult livers). e, UMAP visualization of HLCs transduced with transcription factors NFIX, NFIA and GFP (control) showing that TFs can increase ALB expression while decreasing the expression of the fetal marker AFP. f, Heatmap showing the acquisition of functional hepatocytes markers in transduced hepatocytes derived from hPSCs (*n*=1 sample sequenced per transduction). Heatmap colour scales show “gene expression [mean-scaled, log-normalized counts]”.

To further exploit our single-cell map and the data generated above, we addressed the challenge of cellular maturation associated with human pluripotent stem cell (hPSC)^46^ differentiation. It is well established that most protocols currently available to differentiate hPSCs result in cells with fetal characteristics^47^ rather than fully functional, adult-like cells. Accordingly, single-cell analyses and detailed characterisations have demonstrated the fetal identity of hPSC-derived hepatocytes^48,49^, however, the mechanisms blocking progress toward an adult phenotype remain unclear. To address this question, we performed scRNA-seq on hPSCs differentiating into hepatocyte-like cells (Fig. 7a). PCA analyses confirmed the progressive process driving the acquisition of a hepatocytic identity (Fig. 7b,c). This differentiation trajectory was then compared to the developmental trajectory of primary hepatoblasts/hepatocytes by diffusion pseudotime alignment (Fig. 7d), UMAP, PAGA analyses and PCA (Extended Data Fig. 5a,b). These comparisons showed that HLCs at day 14 of differentiation aligned to the second hepatoblast stage, after which their differentiation follows an *in vitro* specific process. Differential gene expression analyses yielded a list of genes related to xenobiotic metabolism, bile acid transport, and lipid metabolism pathways, which suggests that the divergence between HLCs and primary cells prevents the acquisition of fully adult function. Importantly, a similar divergence from primary development was observed in other cell types generated from hPSCs including cholangiocytes^50^, endothelial cells^32,51^, stellate cells^33^ and macrophages^52^ (Extended Data Fig. 5c-f). Thus, hPSC differentiation could systematically deviate from a natural developmental path after embryonic stages preventing the production of adult cells.

Comparison of *in vivo* to *in vitro* hepatocyte differentiation also revealed transcription factors expressed in fetal hepatocytes (FH1) that were missing in hPSC-derived cells (Extended Data Fig. 6a-c). To validate their functional relevance, these factors were overexpressed in hPSCs differentiated into HLCs for 15 days. The phenotype of the resulting cells was assayed by scRNA-seq 8 days after transduction (Extended Data Fig. 6d). Of particular interest, NFIX or NFIA expression changed the transcriptional profile of HLCs (Fig. 7e,f). This shift was characterised by a decrease in fetal markers and the induction of markers indicative of the adult state (ALB, HP, C3; Fig. 7e-f). Furthermore, pathway enrichment analyses showed an increase in specific functions associated with adult hepatocyte identity, including metabolic and complement-related pathways (Extended Data Fig. 6e). Thus, NFIX or NFIA overexpression during differentiation of hiPSCs appear to increase the expression of specific markers likely downstream of these transcription factors. These results were further validated by inducing the expression of NFIX, NFIA, and CAR during differentiation of hPSCs toward HLCs using the Opti-OX system^53,54^. Stable induction of NFIX, and to a lesser extent NFIA, upregulated an array of functional markers including ALB, SAA1, SAA2, LRP1, CES2, AOX1 while downregulating AFP (Extended Data Fig. 7). Taken together, these results show that mis-expression of key developmental regulators during HLC differentiation could explain their limited capacity to become adult hepatocytes, and that expression of these factors at an early step of differentiation could augment their similarity to adult cells.

## Discussion

Our study provides a detailed map of human liver development and shows that the developmental trajectory of liver cells is influenced by changes in liver environment. Colonisation by the haematopoietic system^55^ appears to initiate the functional maturation of hepatocytes, which progressively acquire intrinsic hepatic functions. Of note, the activation of the liver after birth^24,56^ represents another key change allowing the full maturation of cells. However, this stage could not be included in our analyses since collection of neonatal tissue in human are extremely rare and ethically problematic. Nonetheless, our results established that the step-by-step differentiation of hepatocytes and cholangiocytes originates from a crosstalk among parenchymal and non-parenchymal cells. Of particular interest, stellate cells appear to have an underestimated importance in supporting hepatoblast self-renewal while building the hepatic niche. Such key developmental roles could be shared by many tissue specific fibroblasts involved in organ fibrosis^57,58^ during chronic diseases. In addition, hepatoblasts/hepatocytes are likely to also influence the surrounding cells to establish their own niche and to direct their functional maturation through interactive feedback loops. Utilisation of this knowledge has enabled us to develop a culture system to grow hepatoblasts *in vitro* which provide a promising model system not only to study liver organogenesis, but also to produce cells for clinical applications. Nonetheless, it is important to note that further investigations are necessary to demonstrate the capacity of HBO to differentiate into fully functional hepatocytes and cholangiocytes after clonal isolation. Finally, our developmental map has revealed that differentiation of hPSCs diverge from a natural path of development at an early stage and then follow an *in vitro*-specific process. This divergence explains the fetal nature of cells generated by current protocols^59–64^ and suggests that improving the intermediate, specification steps of these protocols may be necessary to generate adult cells. Thus, understanding organ development remains the best approach for generating fully functional cell types *in vitro*. Our study illustrates how single-cell analyses can be combined with *in vitro* models to uncover the mechanisms driving the generation of functional hepatic cells *in vivo* and thus paves the way toward identifying factors for improving differentiation *in vitro*. This methodology and the resulting knowledge are likely to be transferable to other organs and will be useful for generating a diversity cell types for disease modelling and cell-based therapies.

## Methods

### General statement on experimental design

Data collection and analysis were not performed blind to the conditions of the experiments. In addition, there was no randomization in the organization of the experimental conditions or stimulus presentation.

### Adult human liver collection and dissociation

Liver samples were obtained under sterile conditions from deceased transplant organ donors as rapidly as possible after cessation of circulation. Tissue samples were transferred to the laboratory at 4°C in University of Wisconsin (UW) organ preservation solution. Biopsy tissue was taken from the patient, placed in room temperature HepatoZYME-SFM media and processed immediately. Protocols were developed from information in a previously published dissociation method^13^. The liver tissue was washed twice with warm DPBS with Ca^+2^Mg^+2^ + 0.5 mM EDTA, transferred into a petri dish and diced into small pieces (roughly 1 cm^2^ for resections and 0.25 cm^2^ for biopsies) using a scalpel. The tissue was distributed evenly into multiple GentleMacs Tissue Dissociation C Tubes, or a single 1.5 ml Eppendorf tube for biopsies. 37 °C 0.2 Wünsch/ml Liberase enzymatic digestion solution reconstituted in HepatoZYME media (without growth factors) containing DNAse I (2000 U/ml) was added to each tube: 5 ml per tissue dissociator C tube and 1 ml per Eppendorf tube. The enzymatic digestion occurred in an incubating shaker at 37 °C and 200 RPM for 30 mins. The partially degraded extracellular tissue matrix was mechanically dissociated by running two “B” cycles using the “C tube” in the Miltenyi Biotec GentleMACS tissue dissociator. A 1:1 ratio of 20% FBS to 80% DPBS was added to terminate the enzymatic reaction and the cell suspensions were filtered through 70 um filters, with large pieces gently mashed through the filter. Cells dissociated from donor resections were centrifuged at 50 x g, 4 °C for 5 mins to pellet the hepatocyte fraction, followed by centrifugation of the supernatant at 300 x g, 4 °C for 5 mins and subsequently at 650 x g, 4 °C for 7 mins to collect nonparenchymal cell fractions. The hepatocytes were pooled in 5 ml of DPBS, pipetted onto cold 25% percoll solution and centrifuged at 1250 x g, 4 °C for 20 mins without brake. The purified hepatocyte pellet was incubated for 10 mins in 5ml of red blood cell (RBC) lysis solution (Miltenyi Biotec 130-094-183) and pelleted by centrifuging at 50 x g, 4 °C for 5 mins, yielding a cleaned hepatocyte fraction that was resuspended in cold HepatoZYME-SFM for scRNA-seq. The remaining two NPC fractions were pooled together in 3.1ml of DPBS, to which 900μl of debris removal solution is added. The Miltenyi Biotec Debris Removal (Miltenyi Biotec 130-109-398) protocol was followed according to manufacturer’s guidelines to yield a clean cell pellet. The cell pellets were incubated for 7-10 mins in 2 ml of RBC lysis buffer at room temperature and pelleted by centrifuging at 650 x g, 4 °C for 5 mins, yielding a clean NPC fraction that was resuspended in cold HepatoZYME-SFM. Non-parenchymal cell types were isolated from liver biopsies by first centrifuging the total cell suspension at 400 x g, 4 °C for 5 mins and incubating for 10 mins in RBC lysis solution room temperature. Debris removal solution was used to clean remaining debris and dead cells according to manufacturer’s instructions. The clean pelleted cells were resuspended in cold HepatoZYME-SFM media for analyses.

### Fetal human liver collection and dissociation

Primary human fetal tissue was obtained from patients undergoing elective terminations (ethical approval obtained from East of England - Cambridge Central Research Ethics Committee REC-96/085). The liver was dissected from the abdominal cavity and placed into a solution containing Hanks’ buffered saline solution (HBSS) supplemented with 1.07 Wünsch units/ml Liberase DH (Roche Applied Science) and 70 U/ml hyaluronidase (Sigma-Aldrich), and placed on a microplate shaker at 37 °C, 750 RPM, for 15 mins. The sample was subsequently washed three times in HBSS using centrifugation at 400 x g for 5 mins each. The single-cell suspension was then sorted for EPCAM/CD326 positive cells using CD326 microbeads (Miltenyi Biotec 130-061-101), according to the manufacturer’s guidelines.

### Establishment of hepatoblast organoids

The single cell suspension was resuspended in the hepatoblast organoid media (HBO-M); Advanced DMEM/F12 supplemented with HEPES, penicillin/streptomycin and glutamax, 2% B27, 20mM Nicotinamide, 2mM n-acetlycysteine, 50% WNT3A conditioned medium, 10% R-Spondin, 50ng/ml EGF, and 50uM A83-01. For long term culture (greater than 4 passages) 10uM Y27632 was required. To the resuspended cells was added a volume of Growth Factor Reduced Phenol Free Matrigel (Corning) to make the final solution up to 55% Matrigel by volume, and the mixture pipetted into 48 well plates (20uL per well). The plates were placed at 37°C for fifteen minutes to allow the mixture to set, and subsequently 200uL of fresh HBO-media applied to each well.

### Establishment of biliary organoids (BO) and differentiation

Biliary organoids were derived from samples taken from the livers of adult human deceased donors (National Research Ethics Committee East of England – Cambridge South 15/EE/0152). These were maintained and differentiated as per author’s guidelines^39,40^.

### HBO differentiation into hepatocytes

Hepatocyte differentiation medium was made using complete HepatoZYME (Life Technologies 17705-021). This media was comprised of basal HepatoZYME supplemented with nonessential amino acids (ThermoFisher 11140050), chemically defined lipid concentrate (ThermoFisher 11905031), L-glutamine (2 mM), insulin (14 ng/ml), and transferrin (10 ng/ml). To this media was added either oncostatin-M (Sigma Aldrich) at a concentration of 20 ng/ml, EPO (R&D systems) at a concentration of 50 U/ml, or VEGFA (R&D systems) at a concentration of 50 ng/ml. This supplemented Hepatozyme was applied to HBO for seven days, applying fresh medium every 48 hours.

### HBO differentiation into cholangiocytes

HBO Maintenance medium was supplemented with TGF-β (2 ng/ml) and applied to established HBO lines for a total of seven days, applying fresh medium every 48 hours.

### Cytochrome P450 enzyme activity

Cytochrome P450 enzymatic activity was assessed using Promega® P450-Glo™ assay systems using Luciferin-IPA for and Luciferin-PFBE as surrogate markers for cytochrome P450 3A4 and cytochrome p450 3A5/7 respectively. Each substrate was diluted 1:1000 in freshly applied media as per manufacturer’s guidelines and placed on organoids in three-dimensional culture. After incubation at 37°C for four hours the media was collected and 50μl placed into a detection plate with 50μl detection reagent. The solution was then left for twenty minutes prior to being read via a luminometer. Readings were adjusted for the average of three control readings, with the control consisting of the medium and reagents that was kept at 37°C for four hours without contact with cells.

### Media protein analysis

Albumin, alpha-fetoprotein, apolipoprotein-B, and alpha-1-antitrypsin were detected in media by enzyme linked immunosorbent assay (performed by core biomedical assay laboratory, Cambridge University Hospitals). Concentrations were normalized to cell number.

### Murine blood protein analysis

Blood was drawn retro-orbitally for human albumin ELISA (Bethyl Laboratories) immediately prior to sacrifice at the termination of the experiment (27 days). Serum was separated by centrifugation and levels of human albumin were determined by an enzyme-linked immunosorbent assay (ELISA) using goat polyclonal capture and horseradish peroxidase–conjugated goat anti-human albumin detection antibodies (Bethyl Laboratories). Non-implanted FNRG mouse blood serum was included as a negative control.

### Implantation of HBO and induction of liver injury in mice

All surgical procedures were conducted according to protocol 4388-01 approved by the University of Washington Institutional Animal Care and Use Committees. 7 female FRGN (Fah−/−, Rag2−/−, Il2rg−/−, on a NOD background) mice aged 14 to 18-weeks were used for implant procedures. 14 to 18-week-old female Fah−/− backcrossed to NOD, Rag2−/−, and Il2rg-null (FNRG) mice (Yecuris) were administered sustained release buprenorphrine and anesthetized with isofluorane. Three organoid tissues were sutured onto the inguinal fat pads of each mouse. Three mice received organoid tissues with Donor 4 hepatoblasts and 2 mice received organoid tissues with Donor 5 hepatoblasts. Incisions were closed aseptically. Nitisinone (NTBC) was withdrawn from animals’ drinking water immediately after implantation of organoid tissues and for 14 days after implantation to induce liver injury. NTBC was then reintroduced to the drinking water to allow for recovery, and then removed again after 4 days for the remainder of the experiment. Animals were sacrificed 27 days after implantation of organoid tissues. No statistical methods were used to pre-determine sample sizes as this was not relevant for our study; our sample sizes are similar to those reported in previous publications^38^.

### Immunostaining of harvested constructs from mice

Implants were harvested and fixed in 4% paraformaldehyde for 48 hours at 4°C. Excess fat was trimmed off of the implants, which were then dehydrated in graded ethanol (50-100%), embedded in paraffin, and sectioned using a microtome (6 mm). Some sections were histochemically stained with hematoxylin and eosin. For immunostaining, sections were blocked with normal donkey serum and incubated with primary antibodies against human (see Supplementary Tables for antibodies). To semi-quantify KRT19 distribution in nodules, graft nodules in which all cells were KRT18+/KRT19+ were tallied as “+”. Nodules with both KRT18+/KRT19+ and KRT18+/KRT19- cells were tallied as “+/-”. Nodules with only KRT18+/KRT19- cells were tallied as “-”. Nodules in each category were summed across each tissue section and divided by total KRT18+ grafts in the section to acquire percentages in each animal, with each data point representing one animal.

### Fluorescent *in situ* mRNA hybridization

Primary fetal human liver tissue was fixed in 10% formalin and embedded in paraffin (FFPE), sectioned on to slides and stored for smFISH. FFPE slides were baked at 65°c for 1 hour. Slides were deparaffinized with two washes for 10mins each in xylene solution (Bond™ Dewax Solution, Leica AR9222) and two washes for 5 mins each in PBS and air dried.

Multiplex smFISH was performed on a Leica BondRX fully automated stainer, using RNAScope© Multiplex Fluorescent V2 technology (Advanced Cell Diagnostics 322000). Slides underwent heat-induced epitope retrieval with Epitope Retrieval Solution 2 (pH 9.0, Leica AR9640) at 95°C for 5 mins. Slides were then incubated in RNAScope© Protease III reagent (ACD 322340) at 42°C for 15 mins, before being treated with RNAScope© Hydrogen Peroxide (ACD 322330) for 10 mins at RT to inactivate endogenous peroxidases.

All double-Z mRNA probes were designed by ACD for RNAScope on Leica Automated Systems. Slides were incubated in RNAScope 2.5 LS probes (designed against human genes RSPO3, LGR5, ALB, PDGFRB, LRP5, DES, DKK1, EPCAM, NOTCH2, KDR, DLL4, WNT2B and WNT4) for 2 hours at RT. DNA amplification trees were built through consecutive incubations in AMP1 (preamplifier, ACD 323101), AMP2 (background reduction, ACD 323102) and AMP3 (amplifier, ACD 323103) reagents for 15 to 30 mins each at 42°C. Slides were washed in LS Rinse buffer (ACD 320058) between incubations.

After amplification, probe channels were detected sequentially via HRP–TSA labelling. To develop the C1–C3 probe signals, samples were incubated in channel-specific horseradish peroxidase (HRP) reagents for 30 mins, TSA fluorophores for 30 min and HRP-blocking reagent for 15 min at 42 °C. The probes in C1, C2 and C3 channels were labelled using Opal 520 (Akoya FP1487001KT), Opal 570 (Akoya FP1488001KT), and Opal 650 (Akoya FP1496001KT) fluorophores (diluted 1:500) respectively. The C4 probe complexes were first incubated with TSA–Biotin (Akoya NEL700A001KT, 1:250) for 30 min at RT, followed by streptavidin-conjugated Atto425 (Sigma 56759, 1:400) for 30 min at RT. Samples were then incubated in DAPI (Sigma, 0.25μg/ml) for 20 mins at RT, to mark cell nuclei. Slides were briefly air-dried and manually mounted using ~90 μl of Prolong Diamond Antifade (Fisher Scientific) and standard coverslips (24 × 50 mm^2^; Fisher Scientific). Slides were dried at RT for 24 hrs before storage at 4°C for >24 hrs before imaging.

SmFISH stained fetal liver slides were imaged on an Operetta CLS high-content screening microscope (Perkin Elmer). Image acquisition was controlled using Perkin Elmer’s Harmony software. High resolution smFISH images were acquired in confocal mode using an sCMOS camera and x40 NA 1.1 automated water-dispensing objective. Each field and channel were imaged with a z-stack of 20 planes with a 1μm step size between planes. All appropriate fields of the tissue section were manually selected and imaged with an 8% overlap.

### Differentiation of hPSCs into hepatocyte-like cells

The differentiation of hiPSCs to HLCs followed the protocol previously published by the Vallier lab^59,60^. Prior to differentiation, 12-well plates were coated with 0.1% gelatin for an hour at 37 °C followed by MEF medium overnight at 37 °C and washed with DPBS before use. The hiPSC were passaged at 70-90% confluency by incubating at 37 °C for 4 mins with Accutase cell dissociation reagent (ThermoFisher A1110501). The cell suspension was diluted at a 1:1 ratio with complete E8 media (Gibco A1517001), pipetted gently to mechanically break any clumps into single cells, and pelleted by centrifuging at ~350 x g for 3 mins. The hiPSCs were plated at a density of 50,000-60,000 cells/cm^2^ in 12-well plates in complete E8 media supplemented with Y27632 ROCK inhibitor (10 uM) on 0.1% gelatin/MEF-coated plates. The medium was changed the following day to complete E8 without ROCK inhibitor. On day 1 of differentiation (2 days after plating), CDM-PVA media supplemented with activin (100 ng/ml), FGF2 (80 ng/ml), BMP4 (10 ng/ml), LY294002 (10 uM), and CHIR99021 (3 uM) was added to the cells to induce endoderm formation (see Supplementary Tables for all complete media compositions). CHIR99021 was removed from this medium on day 2 of differentiation. On day 3, RPMI-1640 with nonessential amino acids (ThermoFisher 11140050) and B27 supplement (ThermoFisher 17504044) was supplemented with activin (100 ng/ml) and FGF2 (80 ng/ml). Foregut differentiation was initiated on day 4 and carried out until day 8 by changing media to complete RPMI media supplemented with activin (50 ng/ml). The hepatoblast and subsequent hepatocyte phenotype was induced by changing medium to HepatoZYME-SFM (Life Technologies 17705-021) supplemented with nonessential amino acids (ThermoFisher 11140050), chemically defined lipid concentrate (ThermoFisher 11905031), L-glutamine (2 mM), insulin (14 ng/ml), transferrin (10 ng/ml), oncostatin M (OSM) (20 ng/ml) and hepatocyte growth factor (HGF) (50 ng/ml) from day 9 to day 33.

### Differentiation of hPSCs into cholangiocyte-like cells

Differentiation of hPSCs to CLCs followed the protocol previously published in our lab^50^. Prior to differentiation, 12-well plates were coated with 0.1% gelatin for an hour at 37 °C followed by MEF medium overnight at 37 °C and washed with DPBS before use. The hiPSC were passaged at 70-90% confluency by incubating at 37 °C for 4 mins with Accutase cell dissociation reagent (ThermoFisher A1110501). The cell suspension was diluted at a 1:1 ratio with complete E8 media (Gibco A1517001), pipetted gently to mechanically break any clumps into single cells, and pelleted by centrifuging at ~350 x g for 3 mins. The hPSC were plated in complete E8 medium supplemented with ROCK inhibitor Y27632 (10 uM) at a density of 50,000-60,000 cells/cm^2^ on 0.1% gelatin/MEF-coated plates. The media was changed the following day to E8 without ROCK inhibitor, and two days after plating, the differentiation was started by changing the media to CDM-PVA supplemented with activin (100 ng/ml), FGF2 (80 ng/ml), BMP4 (10 ng/ml), LY294002 (10 uM), and CHIR99021 (3 uM) to induce endoderm formation. The same medium was used the following day (day 2) without CHIR99021. On day 3, RPMI-1640 with nonessential amino acids and B27 supplement (ThermoFisher 17504044) (RPMI+ media) was supplemented with activin (100 ng/ml) and FGF2 (80 ng/ml) only. Hepatoblasts were induced from day 9 to day 12 using RPMI+ medium supplemented with SB (10 uM) and BMP4 (50 ng/ml). This bipotent progenitor was directed toward the cholangiocyte lineage from day 13 to day 16 by feeding with RPMI+ media supplemented with retinoic acid (3 uM), FGF10 (50 ng/ml), and activin (50 ng/ml). The mature cholangiocyte phenotype was induced by re-plating the cells in 3D culture using a 1:2 ratio of cell suspension to Matrigel. The cells matured from day 17 to day 26 in 3D culture with common bile duct media (CBD media) supplemented with EGF (50 ng/ml) and forskolin (10 uM), yielding CLCs at day 26 of differentiation.

### Differentiation of hPSC into hepatic stellate-like cells

This differentiation protocol was gathered from a previously published paper^33^. Prior to differentiation, 12-well plates coated with a 1:50 ratio of reduced growth factor Matrigel and low glucose DMEM medium overnight at 37 °C and washed with DPBS before use. The hiPSC were passaged at 70-90% confluency by incubating at 37 °C for 4 mins with Accutase cell dissociation reagent (ThermoFisher A1110501). The cell suspension was diluted at a 1:1 ratio with complete E8 media (Gibco A1517001), pipetted gently to mechanically break any clumps into single cells, and pelleted by centrifuging at ~350 x g for 3 mins. The hiPSC were plated in complete E8 media supplemented with Y27632 (10 uM) on 12-well plates coated with a 1:50 ratio of reduced growth factor Matrigel and low glucose DMEM overnight at a concentration of 90,000 cells/cm^2^. The media was changed to E8 without ROCK inhibitor the following day. The cells were differentiated to mesoderm by adding DMEM-MCDB 201 media supplemented with BMP4 (20 ng/ml) on day 1 and day 3 of differentiation. On day 5, a mesenchymal phenotype was induced by adding DMEM-MCDB 201 media with BMP4 (20 ng/ml), FGF1 (20 ng/ml), and FGF3 (20 ng/ml). These cells transitioned to liver mesothelium by adding DMEM-MCDB 201 supplemented with retinoic acid (RA) (5 uM), palmitic acid (PA) (100 uM), FGF1 (20 ng/ml), and FGF3 (20 ng/ml) on day 7. From days 9 to 13, the cells were fed every 2 days with RA (5 uM) and PA (100 uM) to attain a fetal hepatic stellate-like cell phenotype (HSLC).

### Differentiation of hPSC into endothelial-like cells

This protocol was adapted from previously published papers^32,51^. Prior to differentiation, 12-well plates were coated with 0.1% gelatin for an hour at 37 °C followed by MEF media overnight at 37 °C and washed with DPBS before use. The hiPSC were passaged at 70-90% confluency by incubating at 37 °C for 4 mins with Accutase cell dissociation reagent (ThermoFisher A1110501). The cell suspension was diluted at a 1:1 ratio with complete E8 media (Gibco A1517001), pipetted gently to mechanically break any clumps into single cells, and pelleted by centrifuging at ~350 x g for 3 mins. The hiPSC were plated in E8 media supplemented with Y27632 (10 uM) at a density of 45,000 cells/cm^2^ on 0.1% gelatin/MEF-coated plates. Differentiation was begun the follow day (day 1) by inducing mesoderm using CDM-PVA media supplemented with FGF2 (20 ng/ml), BMP4 (10 ng/ml), and LY294002 (10 uM). On day 2.5, the cells were fed with Stempro-34 media with VEGFA (200 ng/ml), forskolin (2 uM), and L-ascorbic acid (1 mM). The media was changed every day until day 5.5 to yield fetal endothelial-like cells (ELCs).

### Differentiation of hiPSC into macrophage-like cells

This protocol was adapted from a previously published protocol^52^. The hiPSC were passaged at 70-90% confluency and plated onto an ultra-low adherence 96-well plate with embryoid body (EB) media consisting of E8 supplemented with Y27632 (10 uM), BMP4 (50 ng/ml), SCF (20 ng/ml), and VEGF (50 ng/ml). The cells were incubated for 4 days with half of the media in each well being replaced with fresh media after 2 days. On day 4, the EBs were transferred to a 6-well plate coated with 0.1% gelatin in DPBS, and X-VIVO 15 media supplemented with Glutamax (2 mM), 2-mercaptoethanol (55 uM), M-CSF (100 ng/ml), and IL-3 (25 ng/ml) was added to the wells. Every 5 days, for roughly 10 days, 2/3 of the media in each well was changed. On day 14, the EBs began production of macrophage progenitors. These floating progenitors were collected with the supernatant and plated on uncoated dishes in RPMI + 10% FBS supplemented with M-CSF (100 ng/ml) On day 7 after macrophage progenitor plating, the differentiated macrophages were collected for downstream analyses.

### hIPSCs derived stellate-endothelial progenitor cells

Cells were plated and differentiated according to the endothelial-like cell (ELC) differentiation protocol described (see Methods). At day 3.5 of this protocol, the SEpro cells were present in culture and collected to analyses. To test the bipotentiality of these cells, they were dissociated from the palte into a single-cell suspensions by incubating with TryplE for 20 min at 37 °C, resuspending in culture media and replating at a density of 100,000 cells/cm^2^. These cells were subjected to the hepatic stellate-like cell (HSLC) differentiation conditions beginning at day 7 of this protocol and continuing for at least 4 days to produce HSLCs.

### Lentiviral transduction

Lentiviral aliquots were thawed on ice and added to the desired wells of D15 or D23 differentiating HLCs under sterile conditions. Polybrene (PB) was added to a final concentration of 10 ug/ml to each of the wells, and the plates were rocked gently to ensure even distribution of lentivirus on the adherent cells. The cells were incubated at 37 °C for 24 hrs to allow the lentivirus to infect the cells and stably integrate the transgene into the host genome. After 24 hrs, the cells were washed with DPBS and complete HepatoZYME-SFM with OSM (20 ng/ml) and HGF (50 ng/ml) was added to the cells. This washing procedure was repeated again at 48 hrs and 72 hrs post-transduction to ensure the removal of any remaining lentivirus. hiPSC-derived hepatocyte-like cells were transduced on day 15 of differentiation and assayed on day 23/25.

### Single-cell RNA-Sequencing

Single cell-suspensions from primary tissue and *in vitro* culture were loaded onto the Chromium controller by 10X Genomics, which is a droplet-based single-cell capture platform. The individual cells flowed through the microfluidic chip, were lysed and tagged by a bead containing unique molecular identifiers (UMIs) and were encapsulated in an oil droplet. This resulting emulsion was amplified through reverse-transcription, and follows library preparation as dictated by the 10X Genomics manual. The resulting libraries were sequenced on the Illumina HiSeq 4000 platform. These files were aligned to the GRCh38 human genome and pre-processed using the CellRanger 10X Genomics software for downstream analyses.

### Quality control

Cells expressing fewer than 1000 counts, fewer than 500 genes or more than 40% mitochondrial content were excluded. Application of such filter selected a total number of 237978 cells, which is 87% of the raw number of cells. Genes expressed in fewer than 3 cells were filtered out, leaving 29907 genes (89% of the total number of genes). Doublets were identified by applying two doublet prediction methods: Doubletdetection^16^ and Scrublet^17^.

### Pseudotemporal ordering and alignment

Time-related genes were selected as markers in collection time (Wilcoxon-Rank-Sum test, z-score>10). Diffusion pseudotime of time-related genes was derived using the DPT^19^ routine implemented in SCANPY. Comparison of pseudotemporal trajectories was performed within the cellAlign^29^ framework. cellAlign applies dynamic time warping to compare the dynamics of two single-cell trajectories using a common gene set and to identify local areas of highly conserved expression. The algorithm calculates pairwise distances between ordered points along the two trajectories in gene expression space. cellAlign then finds an optimal path through the matrix of pairwise distances which preserves pseudotemporal ordering and minimises the overall distance between the matched cells. We applied cellAlign onto all corresponding pairs of hiPSCs and primary cell types by selecting genes used for calculating diffusion pseudotime both in hiPSCs and primary cell types. Cells whose distances were lower than a 0.25 quantile threshold were annotated as aligned in Fig. 7a.

### CellPhoneDB analyses

The significance of cellular interactions between cell types was calculated with a publicly available repository of curated receptors, ligands and their interactions (CellPhoneDB^43^, v2.0). Normalised data for primary tissues at each collection time point were used as input. Significance of interactions was calculated based on random permutations of cluster labels to generate a null distribution. Interactions were considered significant based on the default p-value threshold (p-value<0.05).

### Statistics and reproducibility

Primary cell-specific stage-identification was determined based on the standard Louvain clustering routine of scRNA-seq data in SCANPY. Calculation of differentially expressed genes was calculated using a p-value threshold (p<0.01) and absolute log-fold change threshold (|log-fold change| > 1.0). Calculation of time-related genes were selected based on z-score (z-score>10) thresholds for statistical significance using the Wilcoxon-Rank-Sum test, as stated above. Diffusion pseudotime was calculated using the SCANPY computational routine. Significant values for CellPhoneDB ligand-receptor interactions were selected based on a p-value threshold for significance (p-value<0.05). Traditional ORA pathway enrichment of the interactions revealed by CellPhoneDB were selected for statistical significance based on a p-value threshold (p<1e-16). Transcriptomic shifts between control hPSC-derived cells and control, untreated cells were determined based on Louvain clustering with standard parameters in SCANPY, as well as through differential gene expression based on a p-value threshold (p<0.01) and absolute log-fold change threshold (|log-fold change| > 1.0).

Statistical tests comparing groups in qPCR analyses were calculated using GraphPad Prism. Unpaired samples were compared for each condition using unpaired, two-tailed t-tests, as annotated in the corresponding figure legends (data points and error bars correspond to mean values ± SEM). Data distribution was assumed to be normal unless stated otherwise, but this was not formally tested. Outlying data points were excluded based known experimental error or statistical significance of an outlier test (P < 0.05). All immunofluorescent and histology stains are representative and correlate to sequencing results, with each micrograph repeated at least twice. All biologically-independent replicates are stated explicitly in their respective figure legends.

### Cell lines

The hiPSC CA1ATD was was published in Yusa & Rashid et al., 2011^60^. The hiPSC lines FSPS13B, YEMZ, and KOLF were produced and extensively characterized by the Wellcome Trust Sanger Institute HipSci initiative. The HEK239T line is commercially available from ATCC (CRL-11268) and the HUVEC line is available from Lonza (C2519A). The human fetal hepatoblast organoid lines were generated and characterized thoroughly in this study. None of the cross-contaminated or misidentified cell lines on the list maintained by the International Cell Line Authentication Committee (ICLAC) were used in this study.

## Extended Data

**Figure:**
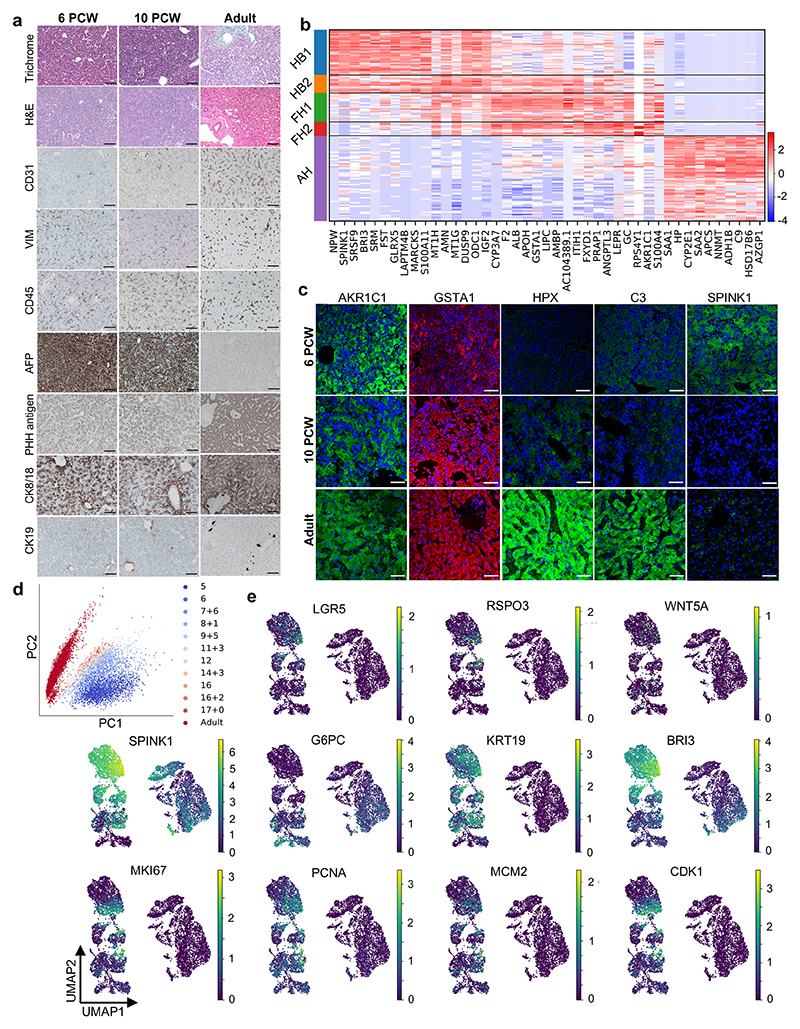


**Figure:**
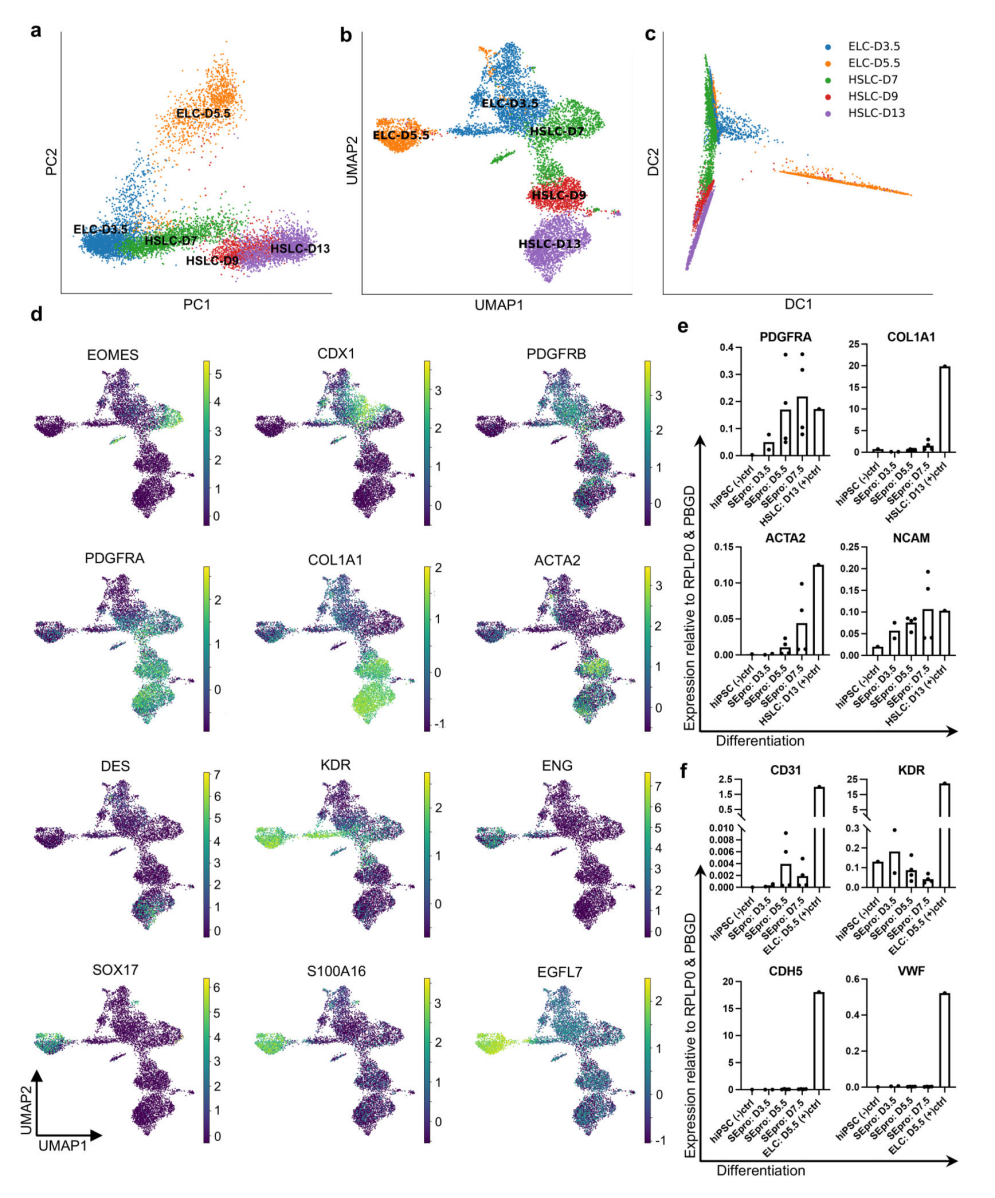


**Figure:**
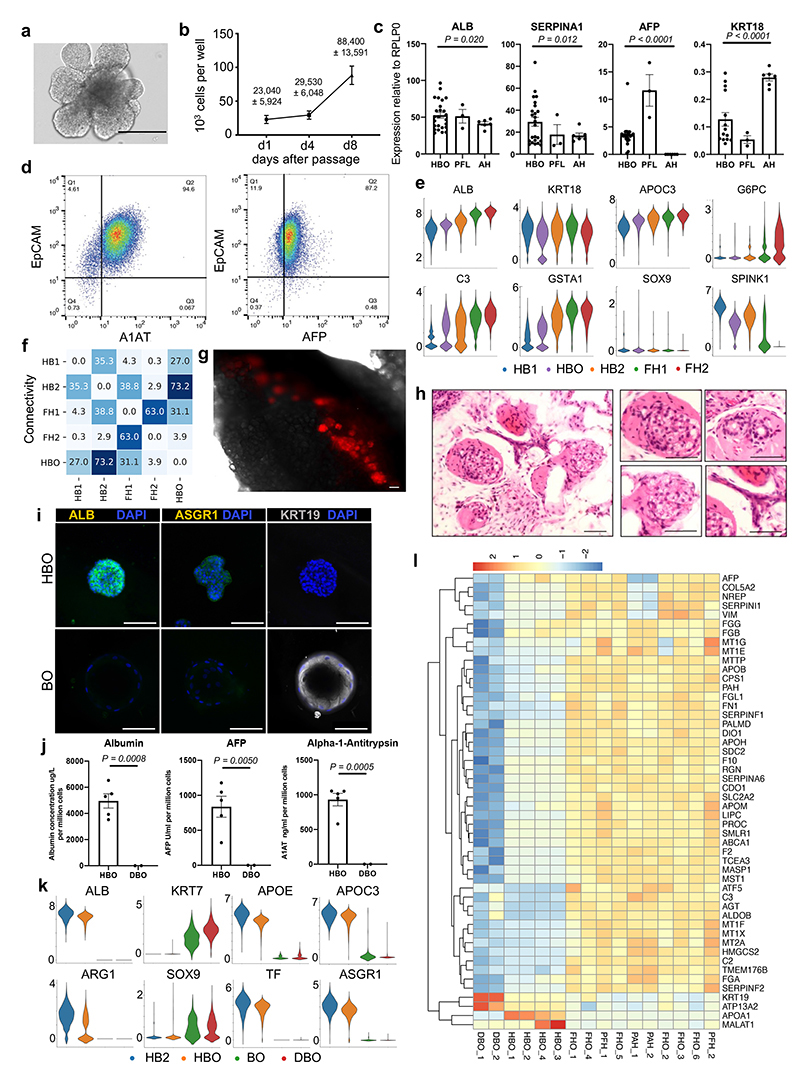


**Figure:**
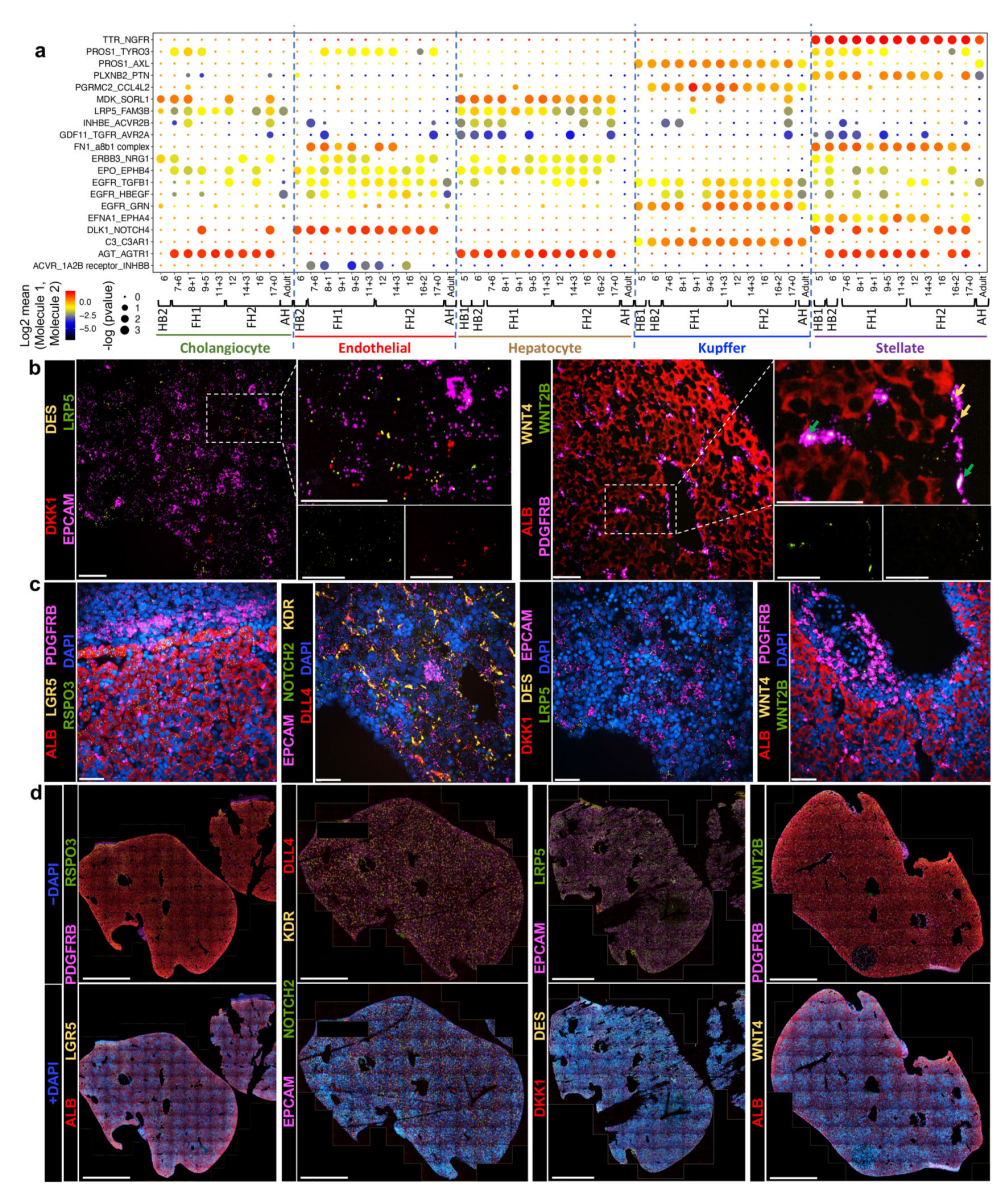


**Figure:**
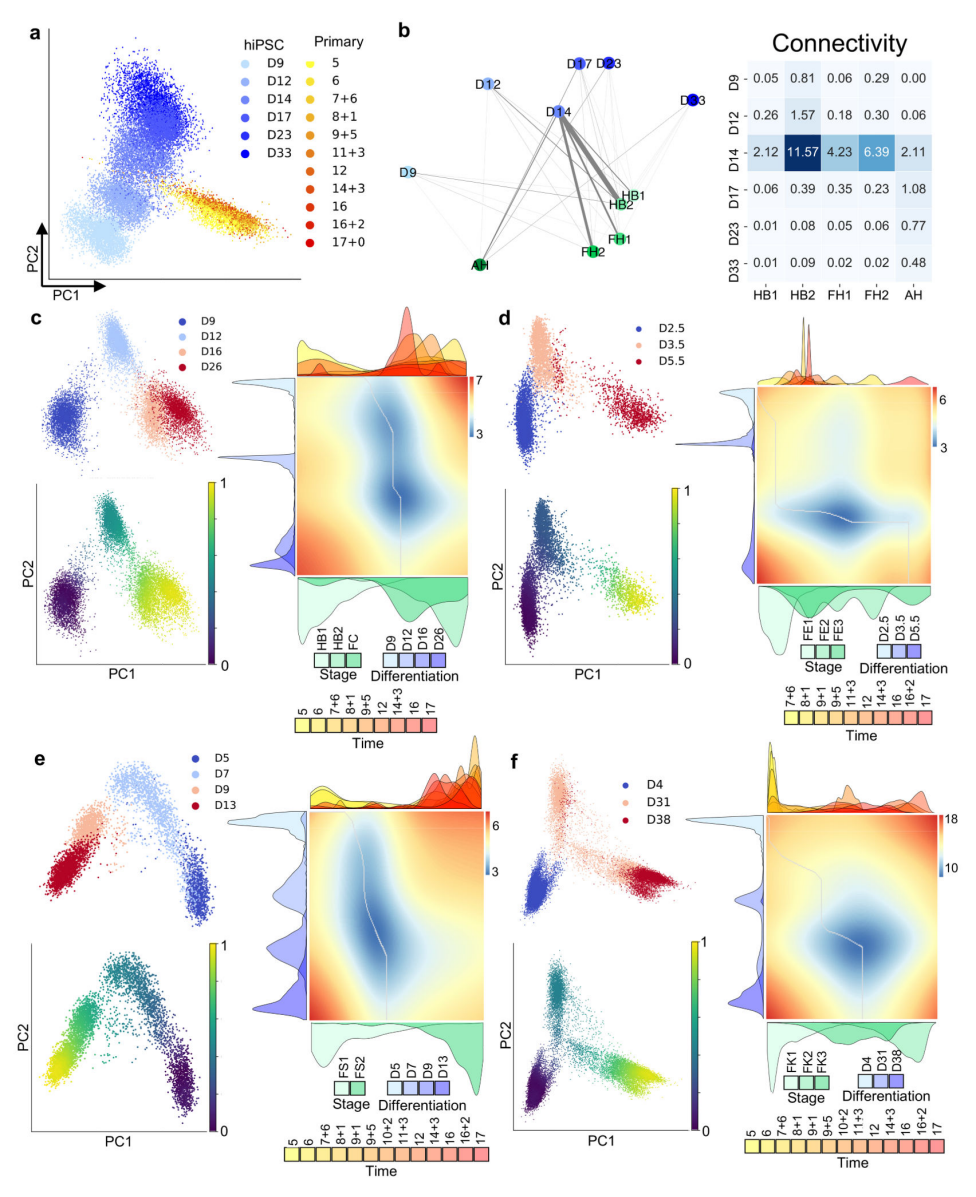


**Figure:**
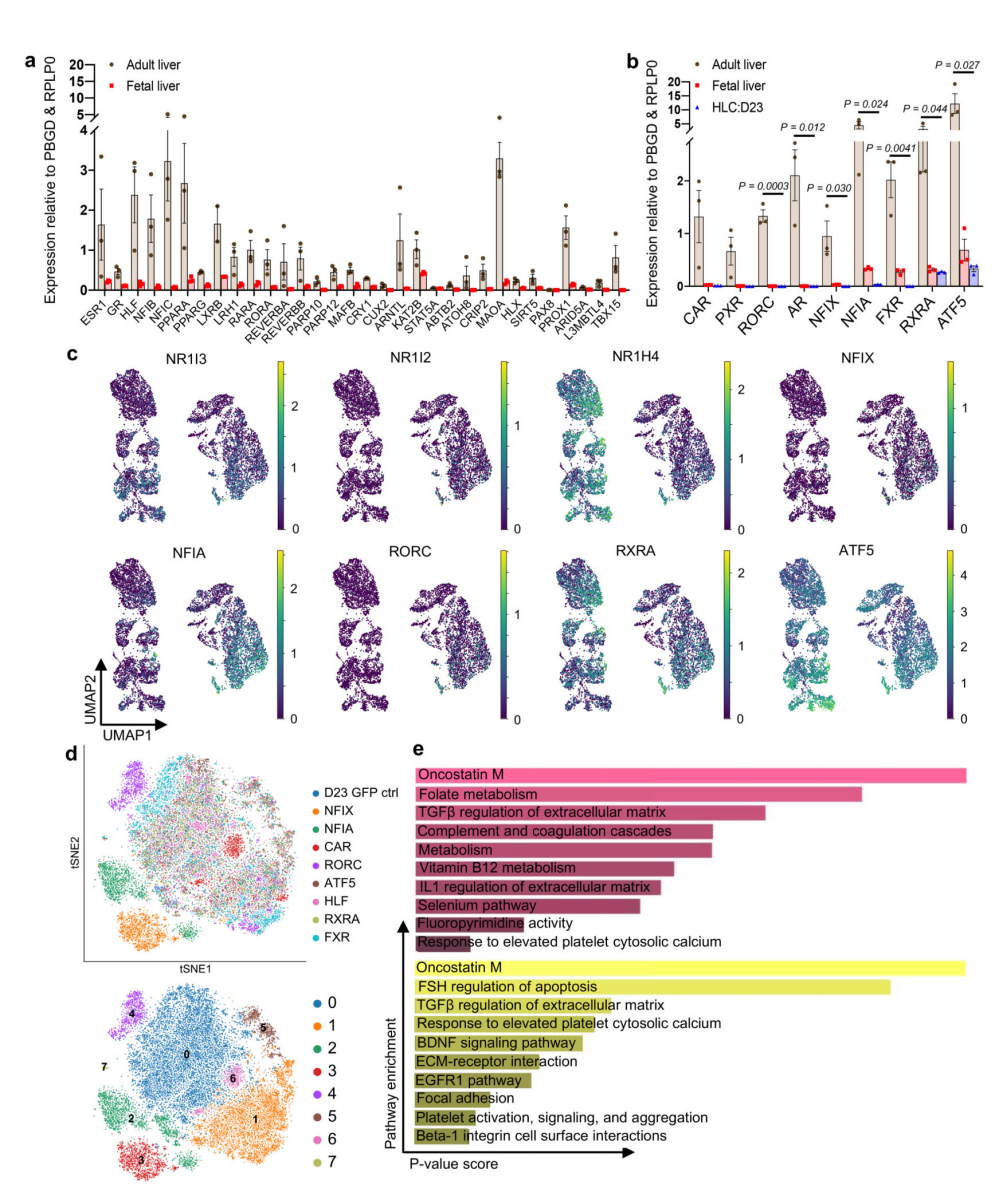


**Figure:**
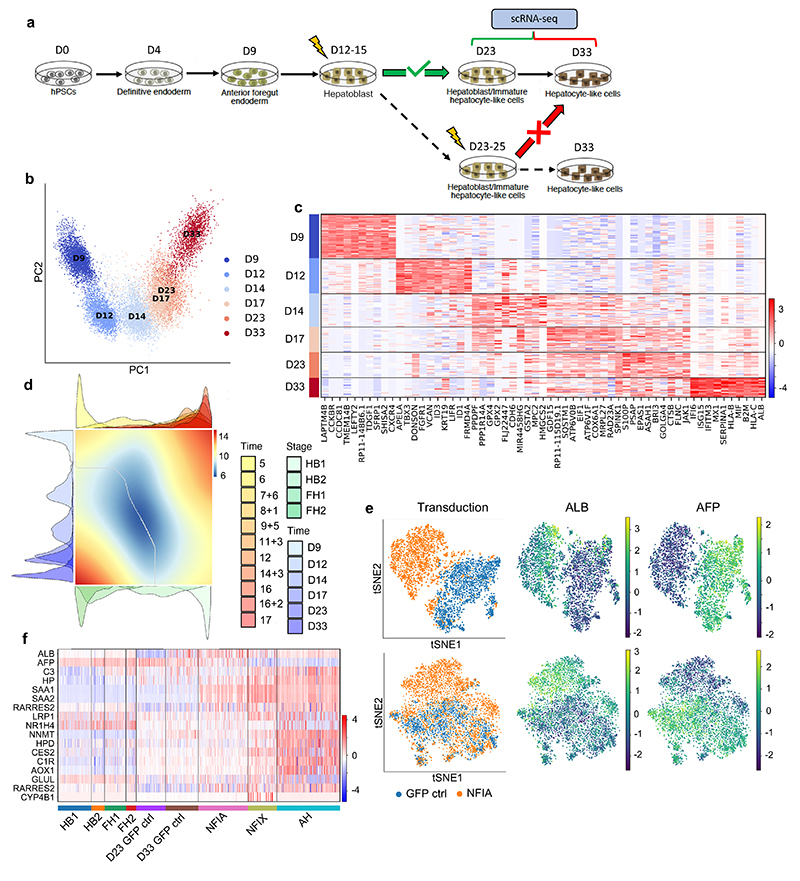


## Supplementary Material

Extended Data Figure 2 Source Data

Extended Data Figure 3 Source Data

Extended Data Figure 5 Source Data

Extended Data Figure 6 Source Data

Extended Data Figure 7 Source Data

Figure 1 Source Data

Figure 2 Source Data

Figure 3 Source Data

Figure 4 Source Data

Figure 5 Source Data

Figure 6 Source Data

Figure 7 Source Data

Supplementary Information

## Data Availability

Sequencing data that support the findings of this study have been deposited in ArrayExpress under accession code E-MTAB-8210. Fetal liver sequencing data has been deposited to ArrayExpress under accession E-MTAB-7189. Previously published fetal liver scRNA-seq data from Popescu et al. has been deposited in ArrayExpress under accession E-MTAB-7407. Previously published adult liver scRNA-Seq data from MacParland et al. and Ramachandran et al. have been deposited in NCBI GEO under accession GSE115469 and GSE136103, respectively. All data sources are described in the Supplementary Tables. All additional raw numerical source data presented in plots and graphs in this study are found in the Source Data files. Any additional data is available upon reasonable request.

## Code availability

All Python and R scripts supporting the findings of this paper are available upon reasonable request.
