# Supplementary material for: Single-cell atlas of human liver development reveals pathways directing hepatic cell fates
Source: Nat Cell Biol. Author manuscript; Available in PMC 2024 Dec 7. (PMC7617064; doi:10.1038/s41556-022-00989-7)
Supplement: Supplementary Information [file EMS151900-supplement-Supplementary_Information.pdf]

Supplementary Table 1 | Sample collection summary. Description of samples used in this study and information on the accessibility/availability of data generated.

scRNA-sequencing Samples	Age/Source	Dissociation method	Accession #	Reference
Adult human liver	Adult	Enzymatic & mechanical Percoll cleanup	E-MTAB-8210	Unique dataset in this manuscript
Adult human cholangiocytes	Adult	Enzymatic & mechanical EPCAM+ MACS	E-MTAB-8495	Dataset generated in collaboration (Sampaziotis et al., Science, 2020)
Fetal human liver	5 PCW	Enzymatic EPCAM+ MACS	E-MTAB-8210	Unique dataset in this manuscript
Fetal human liver	6 PCW	Enzymatic EPCAM+ MACS	E-MTAB-7189	Unique dataset in this manuscript
Fetal human liver	7-17 PCW	Enzymatic CD45+/- MACS	E-MTAB-7407	Popescu et al., Nature, 2019
Adult human liver	Adult	Enzymatic perfusion	GSE115469	MacParland et al., Nature Communications, 2018
Adult human liver	Adult	Enzymatic & mechanical	GSE136103	Ramachandran et al., Nature, 2019
hiPSC-derived hepatocytes	HLC (hiPSC)	Enzymatic (cell culture)	E-MTAB-8210	Unique dataset in this manuscript
hiPSC-derived cholangiocytes	CLC (hiPSC)	Enzymatic (cell culture)	E-MTAB-8210	Unique dataset in this manuscript
hiPSC-derived stellate cells	HSLC (hiPSC)	Enzymatic (cell culture)	E-MTAB-8210	Unique dataset in this manuscript
hiPSC-derived endothelial cells	ELC (hiPSC)	Enzymatic (cell culture)	E-MTAB-8210	Unique dataset in this manuscript
hiPSC-derived macrophages	MLC (hiPSC)	Enzymatic (cell culture)	E-MTAB-8210	Unique dataset in this manuscript (generated by Alsinet-Armengol et al.)
Transcription factor overexpressions during HLC differentiation	HLC (hiPSC)	Enzymatic (cell culture)	E-MTAB-8210	Unique dataset in this manuscript

Supplementary Table 2 | Primary cell type quantification. Description of all primary cell types used throughout this study and their respective cell counts.

time (PCW+days)	Hepatocyte	Cholangiocyte	Kupffer	Endothelial	Stellate
**5**	1933	1934	94	-	21
**6**	815	835	461	2	169
**7+6**	287	9	4304	606	372
**8+1**	440	7	5991	582	653
**9+1**	-	-	50	62	17
**9+5**	445	9	10002	386	442
**10+2**	-	-	38	4	35
**11+3**	15	1	1521	114	21
**12**	358	-	3046	63	100
**14+3**	102	3	5029	191	11
**16**	18	11	1274	135	4
**16+2**	20	-	1156	201	37
**17+0**	180	15	2293	277	46
**Adult**	3719	955	3425	985	109

Supplementary Table 3 | Cultured cell type quantification.
Description of all cultured cell types used throughout this study and their respective cell counts.

**Organoids**	Hepatoblast organoid (HBO)	3375
	Differentiated hepatocyte HBO (HBO+HZ)	2473
	Differentiated cholangcioyte HBO (HBO+TGFB)	6705
	Biliary organoid (BO)	126
	Differentiated biliary organoid (DBO)	897
**hiPSC-derived cholangiocyte-like cells (CLCs)**	CLC: D9	3852
	CLC: D12	3556
	CLC: D16	2415
	CLC: D26	2568
**hiPSC-derived endothelial-like cells (ELCs)**	ELC: D2.5	3381
	ELC: D3.5	2402
	ELC: D5.5	1108
**hiPSC-derived hepatocyte-like cells (HLCs)**	HLC: D9	3848
	HLC: D12	3025
	HLC: D14	2884
	HLC: D17	2183
	HLC: D23	2193
	HLC: D33	1707
**hiPSC-derived hepatic stellate-like cells (HSLCs)**	HSLC: D5	924
	HSLC: D7	1488
	HSLC: D9	1141
	HSLC: D13	1952
**hiPSC-derived macrophage-like cells (MLCs)**	MLC: D4	9079
	MLC: D31	6326
	MLC: D38	4987

Supplementary Table 4 | Markers for cell type identification. List of markers used to identify each cell type of interest during human liver development captured in the single-cell RNA sequencing datasets.

Hepatocyte/hepatoblast	Cholangiocyte	Kupffer cell	Endothelial cell	Stellate cell
ALB	KRT8	FCGR3A	PECAM1	COL1A1
AFP	KRT19	CD68	STAB2	DCN
AHSG	KRT7	MARCO	KDR	ACTA2
TTR	SPP1	IRF7	OIT3	GFAP
SERPINA1	OPN3	SPIC	IL1A	LRAT
APOA1	SOX9	CD14	F8	HAND2
GLUL	HNF1B	TLR4	GNG11	PDGFRB
ASL	PROM1	FCGR1A	LDB2	RBP1
CYP2E1		FCGR1G	PPAP2B	ECM1
ASS1		MSR1	GPR116	
PCK1		CETP	FCN3	
G6PC		CSF1R	HES1	
FABP1		TLF9	CRHBP	
RBP4		CLEC4F	STAB1	
FGG			DNASE1L3	
FGA			DLC1	
FGB			CLEC4G	
APOC3				
APOA4				
GSTA1				

Supplementary Table 5 | Media compositions. Detailed description of media and their compositions used for cell culture.

Medium	**Reagent**	**Stock conc.**	**Final amount/conc.**
**CDM-PVA**	Ham's F12 Nutrient Mixture	-	250 ml (49% v/v)
	Iscove's Modified Dulbecco's Media (IMDM)	-	250 ml (49% v/v)
	Chemically defined lipid concentrate	100x	5 ml (1% v/v)
	Penicillin/streptomycin	100x	5 ml (50-100 U/ml)
	1-thioglycerol (MTG)	-	20 ul (1 * 10-8% v/v)
	Insulin	10 mg/ml	250 ul (7 ng/ml)
	Transferrin	10 mg/ml	350 ul (5 ng/ml)
	Polyvinyl alcohol (PVA)	-	0.5 g (0.1% w/v)
**RPMI+**	RPMI-1600	-	480 ml (96% v/v)
	Nonessential amino acids (NEAA)	100x	5 ml (1% v/v)
	Penicillin/strepotomycin	100x	5 ml (50-100 U/ml)
	B27 plus insulin supplement	50x	10 ml (2% v/v)
**HepatoZYME+**	HepatoZYME basal media	-	470 ml (94% v/v)
	Nonessential amino acids (NEAA)	100x	10 ml (2% v/v)
	Chemically defined lipid concentrate	100x	10 ml (2% v/v)
	Penicillin/streptomycin	100x	5 ml (50-100 U/ml)
	L-glutamine	200 mM	5 ml (2 mM)
	Insulin	10 mg/ml	700 ul (14 ng/ml)
	Transferrin	10 mg/ml	500 ul (10 ng/ml)
**Common bile duct**	William's E media	-	480 ml (96% v/v)
	Nicotinamide	0.4 M	12.5 ml (10 mM)
	HEPES buffer	1 M	10 ml (20 mM)
	Sodium bicarbonate (NaHCO3)	1 M	8.5 ml (17 mM)
	Sodium pyruvate (C3H3NaO3)	100 mM	3.15 ml (6.3 mM)
	L-ascorbid acid 2-phosphate (AA2P)	100 mM	1 ml (0.2 mM)
	Glucose	1 M	7 ml (14 mM)
	Insulin-Transferrin-Selenium (ITS)	100x	5 ml (1% v/v)
	Dexamethasone	10 mM	5 ml (0.1 uM)
	Glutamine	190 mM	5.3 ml (2 mM)
	Penicillin/streptomycin	100x	5 ml (50-100 U/ml)
**DMEM-MCDB 201**	DMEM low glucose	-	288.5 ml (~57% v/v)
	MCDB-201-water	-	202.5 ml (~40% v/v)
	LA-BSA	100x	1.25 ml (0.25x)
	Insulin-Transferrin-Selenium (ITS)	100x	1.25 ml (0.25x)
	Penicillin/streptomycin	100x	5 ml (50-100 U/ml)
	Ascorbic acid/Vitamin C	250 mM	200 ul (0.1 mM)
	Dexamethasone	1.5 mM	835 ul (2.5 uM)
	2-mercaptoethanol	50 mM	500 ul (50 uM)
**Essential 8 (house)**	DMEM/F-12	-	500 ml (95.5% v/v)
	Insulin-Transferrin-Selenium (ITS)	100x	10 ml (2x)
	Sodium bicarbonate (NaHCO3)	7.5% w/v	3.6 ml (1.74 g/L)
	L-ascorbid acid 2-phosphate (AA2P)	6.41 mg/ml	5 ml (61.2 ug/ml)
	Penicillin/streptomycin	100x	5 ml (50-100 U/ml)
**Essential 8 (commerical)**	E8 basal media (ThermoFisher A1517001)	-	500 ml (97% v/v)
	Nutrient supplement	-	10 ml (2% v/v)
	Penicillin/streptomycin	100x	5 ml (50-100 U/ml)

Supplementary Table 6 | Immunochemistry antibodies. List of antibodies used throughout this study.

Antibody	Gene	Cell type	Species	Dilution	Application	Source	Identifier	Clonality
SPINK1	SPINK1	Hepatoblast	Rb	1:200	IHC-P	Abcam	ab207302	Mono
Hemopexin	HPX	Hepatocyte	Rb	1:100-1:250	ICC/IF, IHC-P	Abcam	ab124935	Mono
C3	C3	Hepatocyte	Rb	1:100-1:1000	ICC/IF, IHC-P	Abcam	ab97462	Poly
AKR1C1	AKR1C1	Hepatocyte	Rb	7.5 ug/ml	IHC-P	Abcam	ab192785	Poly
GSTA1	GSTA1	Hepatocyte	Go	1:1000	ICC/IF, IHC-P	Abcam	ab53940	Poly
Cytokeratin 7	KRT7	Cholangiocyte	Rb	1:200	IHC-P, ICC	Abcam	ab68459	Mono
Cytokeratin 19	KRT19	Cholangiocyte	Mo	1:200	IHC-P/Fr, ICC	Abcam	ab7754	Mono
α1-AT	SERPINA1	Hepatocyte	Rb	NA	IHC-P	Dako	A0012	Poly
CD31	PECAM1	Endothelial	Mo	NA	IHC-P	Leica	PA0414	Mono
CD45	PTPRC	Immune cells	Mo	NA	IHC-P	Dako	M070101	Mono
CD68 K	CD68	Macrophage/Kupffer	Mo	NA	IHC-P	Dako	M081401	Mono
CK8/18	KRT8/18	Hepatocyte	Mo	NA	IHC-P	Leica	PA0067	Mono
CK19	KRT19	Cholangiocyte	Mo	NA	IHC-P	Leica	PA0799	Mono
Human hepatocyte	NA	Hepatocyte	Mo	NA	IHC-P	Dako	M715801	Mono
Vimentin	VIM	Mesenchyme	Mo	NA	IHC-P	Dako	M702001	Mono
Albumin	ALB	Hepatoblast/ Hepatocyte	Go	1:100	IHC/IF	Bethyl	A50-103A	Poly
Alpha-1-Antitrypsin	SERPINA1	Hepatoblast/ Hepatocyte	Rb	1:100	IHC/IF	Dako	AA0012	Poly
Alpha-Fetoprotein	AFP	Hepatoblast	Rb	1:100	IHC/IF	Dako	GA500	Poly
Arginase 1	ARG1	Hepatocyte	Rb	1:400	IHC/IF	Sigma-Aldrich	SAB5500008	Mono
Cytokeratin-18	KRT18	Hepatocyte/ cholangiocyte	Mo	1:25	IHC/IF	Dako	GA618	Mono
Hepatocyte nuclear factor-4-alpha	HNF4A	Hepatoblast/ hepatocyte	Mo	1:100	IHC/IF	R&D systems	PP-H1415-00	Mono
SRY-box transcription factor 9	SOX9	Cholangiocyte	Rb	1:100	IHC/IF	Abcam	ab185230	Mono

Supplementary Table 7 | RNAScope probes. List of RNAScope probes used throughout this study.

Probe	Channel	Gene	Species	Source	Cat. No.
RNAscope 2.5 LS Probe- Hs-RSPO3-O4	1	RSPO3	Human	ACD	520498
RNAscope 2.5 LS Probe- Hs-LGR5-C2	2	LGR5	Human	ACD	311028-C2
RNAscope 2.5 LS Probe- Hs-ALB-C3	3	ALB	Human	ACD	600948-C3
RNAscope 2.5 LS Probe- Hs-PDGFRB-C4	4	PDGFRB	Human	ACD	548998-C4
RNAscope LS 2.5 Probe- Hs-WNT2B	1	WNT2B	Human	ACD	453368
RNAscope 2.5 LS Probe- Hs-WNT2B-C2	2	WNT4	Human	ACD	429448-C2
RNAscope LS 2.5 Probe- Hs-LRP5	1	LRP5	Human	ACD	421868
RNAscope 2.5 LS Probe- Hs-KDR-C2	2	KDR	Human	ACD	591618-C2
RNAscope 2.5 LS Probe- Hs-EPCAM-C4	4	EPCAM	Human	ACD	310288-C4
RNAscope 2.5 LS Probe- Hs-DES-C2	2	DES	Human	ACD	403048-C2
RNAscope 2.5 LS Probe- Hs-DKK1-C3	3	DKK1	Human	ACD	421418-C3
RNAscope 2.5 LS Probe- Hs-NOTCH2	1	NOTCH2	Human	ACD	488108
RNAscope 2.5 LS Probe- Hs-DLL4-C3	3	DLL4	Human	ACD	603008-C3
RNAscope 2.5 LS Probe- Hs-GDF10	1	GDF10	Human	ACD	506168
RNAscope 2.5 LS Probe- Hs-ERBB3-C2	2	ERBB3	Human	ACD	311948-C2
RNAscope 2.5 LS Probe- Hs-DLK1	1	DLK1	Human	ACD	529968

Supplementary Table 8 | Quantitative real-time PCR primer sequences. List of RT-qPCR primers used throughout this study.

Gene	Forward primer	Reverse primer
ABTB2	GGTTTCCGCATGCTCAACTG	GGCGTCATACCCTGGTCATC
AFP	AGAACCTGTCACAAGCTGTG	GACAGCAAGCTGAGGATGTC
ALB	CCTTTGGCACAATGAAGTGGGTAACC	CAGCAGTCAGCCATTTCACCATAG
AOX1	ATGCCCTTGGTGGTAACCTG	TACTTTGACAGCAGCCCGAA
APOB	GCCATTGCGACGAAGAAAATA	TGACTGTGGTTGATTGCAGCTT
AR	CGCTGAAGGGAAACAGAAGTACC	GTTTCTTCAGCTTCCGGGCTC
ARID5A	CGCCTCTGGAAGAACGTGTA	CCCTGTTCTCCTTAGCCATCT
ARNTL	CCAGCCCATTGAACATCACG	GTGGGGGTTCTCACCAAGAA
ARNTL2	TGACTGACAAGCACAAAGCAG	CCAGGCTCACTATGTCCCAAAA
ATF5	GCTGGGATGGCTCGTAGAC	CGAGAAGGTTGAGGTGGAGAA
ATOH8	CGCCTCCTCCGAGATCAAA	GCACGGCACCTGCTT
C1R	GCCCAGATGCAAGATCAAGG	GGATACGGGCCTTGTAGGTG
CES2	GAATTCGGCTGGCTCATCCC	TGTAGGAGGCAACATCAGCAG
CRIP2	TTCGGACCCAAGGGAGTGA	ACATCATGAGAGCCGCTGTAG
CRY1	GGTGGACAACCGCCTCTAAC	TCAAAACCTAGCTCTTCCAGTG
CUX2	GCATCCAAGGCAGATGAAGT	GGCGGATGGAGCTGTTGAC
CYP3A4	TGTGCCTGAGAACACCAGAG	GTGGTGGAAATAGTCCCGTG
CYP3A7	GAAACACAGATCCCCCTGAA	TCAGGCTCCACTTACGGTCT
CYP4B1	TTGCAGTCAGCGATCTCACTC	CCCTGATGACCTGGTCTGTATG
ESR1	TCCTCTTCAACCCCGATGTG	CCAGGGTTCCAGGACTTCAC
G6PC	GTGTCCGTGATCGCAGACC	GACGAGGTTGAGCCAGTCTC
GDA	TGATGGGTGAACTGGGCAAC	GTGTGCCATCACTGTCTTATTTGT
GLUL	CATGTTTCGGGACCCCTTCC	GTGCCTCAAATTGGTCTCTGC
HLF	CTCGCAAACGCAAGTTCTCT	TTGTCATCCTTCAGGTCATCAG
HLX	CGGCCAGTTCTTCGCATCTC	CAGCATAGGGACCTGGAAACG
HNF1b	GCACCCCTATGAAGACCCAG	GGACTGTCTGGTTGAATTGTCG
HNF4a	CATGGCCAAGATTGACAACCT	TTCCCATATGTTCCTGCATCAG
HNF4G	TTCAGACGCAGCATTCGTAAG	GCTGCCATCAAATGTGCTTCT
HP	AGGGGGAGAAGTGAGCTAGT	GCTCCCAGGGCACTCATCTT
HPD	CAGCAAGATGGGCTTTGAACC	GTGATCGCCCATCTCTTTGTTC
KAT2B	TAGTCACCTGCCAGCAAAAGA	TGCAGTAACACAGCCACCTTG
KRT19	ACGACCATCCAGGACCTGCGG	TCCCACTTGGCCCCTCAGCGTA
KRT7	GATTGCTGGCCTTCGGGGT	TCATCACAGAGATATTCACGGCTC
L3MBTL4	CCCTTATGTCCAGCCAGTTG	GGGATAACCTTGGGGTGCTAT
LGR5	CTCCCAGGTCTGGTGTGTTG	GAGGTCTAGGTAGGAGGTGAAG
LRP1	GAGTCTGCTTCGTGTGCCTA	GTCATTGTCATTGTCGCATCTCC
MAFB	ACCGAGAGGTCCCAGTACAA	AGCAGAGGGGAGGATCTGTT
MAOA	CGCCTTTCCACCAGTATGGA	ACCGCCTAGCAGTCTTTGTC
MAOB	AGCTCTGCTGGACTGAATCTG	TCTTGTTGTGCCTCCACACT
MDFIC	TCACCTGAAGATTGTTGTGTCCA	GATGCCACATGACGCTTGTC
NFIA	CCTCAGGAGACGCTGAAAGA	CATTGGGGTGGGAAGGAATG
NFIB	AGAAATCCCAAGCCAACCATACT	GGTGAACTTGGAGAGGGGTAA
NFIC	TGGCGGCGATTACTACACTT	CGAGATGCCTCCTTCCATGTC
NNMT	AGTTTGGTTCTAGGCACTCTGC	CCCTTCACACCGTCTAGGCA

NPAS2	CGGTGGTCAGTTACGCAGAT	CCGATGGCGAATGACTGGTA
NR0B2 (SHP)	CTTCTGGAGCCTGGAGCTTAG	GGCACATCGGGGTTGAAGAG
NR1D1 (Rev-erb-a)	CCTCAACGTCCAACCCTTCTG	GTGATGACGCCACCTGTGTT
NR1D2 (Rev-erb-b)	TCATGCTTGCGAAGGCTGTA	ACCAAACCGAACAGCATCTCT
NR1H2 (LXRb)	AACCCAGGGCAACAGGAGTAG	CGTGGTAGGAGAGGACATGGT
NR1H4	ACAAAGTCATGCAGGGAGAAAAC	TCCTGAGGCATCCTCTGTTTGT
NR1H4 (FXR)	ACTGAACTCACCCCAGATCAA	TGGTTGCCATTTCCGTCAAA
NR3C1 (GR)	TGCTGTGTTTTGCTCCTGATC	AGATACCTGAAGCCTGTGTAACT
NR3C2 (MR)	AGCACAATTCCAAAGGATGGC	AGAATTCCAGCAGGTCGCTC
NR5A2 (LRH-1)	GCTCTTAATCCTCGACCACATT	CTCCGGCTTGTGATGCTATT
PARP10	TGACTGGCTTTCGGCTCTGT	GCACAACACATGGCAGCTAA
PARP12	CCCACAGGGGACTTCTGAAAG	TGGGATTGCAATATGCCTCTTC
PAX8	CAAGAGGAAAATGGATGACAGTGA	TAGTGCTGCCGCTCAAATGG
PER2	TGGACAGCGTCATCAGGTACT	ACTGTGGCCTTCCGCTTATC
PPARA	CCCTCCTCGGTGACTTATCC	CGGTCGCACTTGTCATACAC
PPARB (PPARD)	CCGGGACAGTGTTGTACAGT	CCTCGTTGGTGCATCTGTCT
PPARG	GAGCCTGCATCTCCACCTTAT	AGAAACCCTTGCATCCTTCACA
PROX1	TCCGACGTAAAGTTCAACAGATG	GATGGCTTGACGTGCGTACT
RARA	ACACTACGAACAACAGCTCAGA	CTTGGCGAACTCCACAGTCT
RARRES2	TGTGAGGCTGGAATTTAAGCTG	GCATTTCCGTTTCCTCCCAT
RORA	AAGGCTGCAAGGGCTTTTTC	TGTTGGCAGCGGTTTCTACT
RPLP0	GGCGTCCTCGTGGAAGTGAC	GCCTTGCGCATCATGGTGTT
RXRA	ACAGCTGCATTCTCCCATCAG	CTGCTGACGGGGTTCATAGG
SAA1	TTCCATGCTCGGGGGAACTA	GATATTCTCTCTGGCATCGGTGA
SAA2	CTACAGCACAGATCAGCACCA	CCTCGCCAAGGAACGAAAAG
SERPINA1	CCACCGCCATCTTCTTCCTGCCTGA	GAGCTTCAGGGGTGCCTCCTCTG
SIRT5	TCGTGGTCATCACCCAGAAC	AAGCTACCATGGATCTCCAGAAG
SLC25A47	GGACACGGTGAAGGTCAGGAT	GGCGGTAGGTGCCAAAAGAC
SOX9	CTCTGGAGACTTCTGAACGAGAG	CCTTGAAGATGGCGTTGGGG
STAT5A	AGGAACATGTCACTGAAGAGGAT	ACTCAAACAGGACTGTGAACT
TBX15	CGCACACTCACCTTCGAAGA	CTTTCTCGGCAGCCCACATT
TSHZ2	AATAGCTCTGCAGGACGAATG	GCAGGATGGTGCAACGAAGA
ZFHX4	GCACCAGGGCTCGTAAATAA	AGTAAGGAGGGACAGGTCATCA
ZNF385B	AGGGGTCAGGACTACAGAACA	TGGTTTCCCTGCAACTCGAT
ZNF619	GTGCACAGGTGGTAAAACCAA	TCAGCAGTCCCTCCACTATC

Supplementary Methods

Immunohistochemistry of primary tissue sections

Primary human liver tissue was either snap-frozen in OCT, or fixed in 10% formalin and embedded in paraffin, sectioned onto slides, and stored for immunohistochemistry. Paraffin and OCT embedded samples were sectioned and placed onto slides for IHC by the tissue bank at Addenbrooke's Hospital, Cambridge, UK. Paraffin embedded sections were deparaffinated and rehydrated by washing 3 times for 10 mins each with xylene followed by 3 washes for 5 mins each with ethanol. Frozen sections were brought to room temperature, followed by heat-induced antigen retrieval (HIER). All sections were washed for 5 mins in tap water, followed by 20 mins of HIER in 90 ^{o}C citrate buffer with pH 6.0 and another 20 mins in the 90 C buffer at room temperature, allowing the buffer to cool ambiently. Sections were washed for 5 mins in tap water and 5 mins with DPBS + 0.1% TritonX-100, and then were blocked for 30 mins using DPBS + 10% donkey serum (DS) + 0.1% TritonX-100. Primary antibodies were diluted in DPBS + 1% DS and 0.1% Triton-X100 and incubated on the tissue sections overnight at 4 °C. The tissue sections were washed 3 times for 5 mins each with DPBS + 0.1% TritonX-100. The secondary antibodies were diluted in 1:1,000 in DPBS + 1% DS and 0.1% Triton-X100 and incubated on the tissue in the dark at room temperature for 1 hr. The sections were washed 3 times for 5 mins each using DPBS, with the second wash containing 1:10,000 dilution of Hoechst counterstain. Fluorescence preservation mounting media and coverslips were placed on the tissue sections and stored at 4 °C prior to immunofluorescent imaging. Fluorescent IHC imaging was performed using the Zeiss LSM 700 confocal microscope and images were analysed using Imagej 1.48k software (Wayne Rasband, NIHR, USA, http://imagej.nih.gov/ij).

Hematoxylin and eosin (H&E), Masson's trichrome, and other histological stains were performed by the histopathology and cytology department at Addenbrooke's Hospital, Cambridge, UK. This department is jointly staffed by National Health Service (NHS) and

University of Cambridge consultants with expertise in histopathology, and which regularly participates in internal and external quality control schemes. Staining was performed using Leica BOND-MAX automated IHC staining system. Histological imaging was performed using the Zeiss Axio Imager M2 and were processed using Zeiss Zen Blue software. All antibodies used are described in detail in the Supplementary Tables.

Maintenance of hepatoblast organoid

The culture medium (HBO-M, see above) was changed every 48-72 hours, and organoids mechanically passaged every 7-10 days. Organoids were passaged by scraping the gel away from the plate, pipetting the resulting solution into 1.5ml tubes, and pipetting the solution up and down to break individual organoids into pieces. If a precise cell number was required, the organoids can alternatively be processed to a single cell solution as described below, and then re-plated at the required dilution in the 55% Matrigel-medium solution.

Dissociation of HBO into single cell

Organoids established in culture were dissociated to single cell suspensions for splitting/ single cell sequencing/ cell counting by first removing the media from each well and replacing with Cell Recovery Solution (Corning). The organoids in the Matrigel were then be scraped off the plate and placed on ice for 30 minutes to remove the Matrigel. The organoids were then washed with PBS and placed in TrypLE (ThermoFisher Scientific) for 15 mins at 37 ^{o}C. Cells were finally washed three times in PBS or basal media.

Imaging organoids

The medium was removed and replaced with 4% Paraformaldehyde solution for 20 minutes at room temperature to fix the organoids in situ, followed by three washes in PBS (each for five

minutes). Fixed organoids could then be stored in PBS for later Immunohistochemistry. Immunostaining was performed by permeabilizing and blocking with 0.3% Triton X-100 (Sigma-Aldrich), and 10% donkey serum (Biorad) for three hours at room temperature, and then incubated overnight at 4 °C, with the primary antibody in 1% donkey serum. Samples were then washed with three one-hour PBS washes, followed by incubation with the fluorescent secondary antibody overnight at 4 °C. The samples were finally washed three times in PBS, with 1:10000 dilution Hoechst 33258 (ThermoFisher Scientific) added to the first wash.

Flow cytometry

Organoids were dissociated to single cell solution as described above. The single cells were then fixed and permeabilised in FixPerm (ThermoFisher Scientific) for 20 minutes, then washed three times in PBS before resuspension in PermWash (ThermoFisher Scientific). Blocking was performed using 10% donkey serum (Biorad) in PBS for 30 minutes. Primary antibodies were diluted 1:100 in 1% donkey serum in PBS and incubated with the cell suspension at room temperature for 1 hour. After three washes in PBS, cells were incubated with secondary antibodies diluted 1:1000 in 1% donkey serum in PBS. Data were collected using Cyan ADP flow cytometer, and analysed using FlowJo X (BD, FlowJo, LLC).

Freezing/ thawing of HBO

Organoids were extracted from Matrigel using cell recovery solution as described above. The organoids were then washed in PBS and resuspended in Cell Culture Freezing Medium (ThermoFisher Scientific), placed in cryogenic vials (ThermoFisher Scientific), and cooled to -80°C using a "Mr. Frosty" Freezing Container (ThermoFisher Scientific). Organoids could then be kept long term in liquid nitrogen. To thaw, cryovials were kept at 37 ^{o}C until the

freezing medium had melted. Organoids were then washed three times in PBS to remove any residual freezing medium, and then re-plated as above.

Assessment of cell proliferation

Organoids were extracted and dissociated to single cells as described above. The cells were then counted using the countess II automated cell counter (Biorad), and the final number adjusted according to the number of wells used and the volume of resuspension.

BODIPY assay

BODIPY 493/503 (ThermoFisher Scientific) was diluted 1:1000 in the organoid culture media and applied to organoids for 30 minutes. After this time the organoids were washed with fresh media and imaged in situ using a fluorescent microscope.

Generation of reporter lines

HBO were dissociated to a single cell suspension as described. Cells were transfected using a lentiviral vector containing tdTomato (rLV. EF1. tdTomato9, Clontech), according to the manufacturer’s guidelines. Following transfection HBO were grown in medium supplemented with puromycin (2 mg/ml) for ten days to select for stably transfected cells.

Culture of HUVEC cell line for transplantation

Primary human umbilical vein endothelial cells (HUVECs, passage 2) were thawed and cultured in EGM-2 media (Lonza). Media was replaced every 48h. Cells were passaged once before implantation in mice.

Encapsulation of HBO for implantation in mice

Hepatoblast organoids were isolated and then suspended in 55% matrigel (Corning) on ice. HUVECs were suspended in 10 mg/ml fibrin hydrogel (human thrombin, Sigma-Aldrich; bovine fibrinogen, Sigma-Aldrich) and mixed with organoids in un-polymerized matrigel at a 20:1 ratio. Cell/fibrin mix containing organoids and HUVECs was polymerized in disks from which 6 mm biopsies were punched to create "organoid tissues". Each 6 mm implant contained approximately 3,500 organoids and 100,000 HUVECs.

Culture of primary hepatocytes

Primary hepatocytes were purchased from Biopredic International (Rennes, France). Hepatocytes were isolated from human liver resects from three donors (two male and one female), and met the manufacturer's quality control requirements in regards to viability, confluency and functionality, which was assessed by Phase I and II dependent enzymatic activity. Cells were purchased as monolayer cultures and maintained in William's E (Gibco) supplemented with 1% Glutamine (Gibco), 1% Penicillin-streptomycin (Gibco), 700 nM Insulin (Sigma) and 50 μM Hydrocortisone (Sigma) for no longer than 48h upon receipt.

Generation and culture of hPSC lines

The human pluripotent stem cell (hPSC) lines used in this study were generated previously in our lab and collaborators' labs. Cells were maintained in defined culture described previously[46,50,59], using Essential 8 media with the provided nutrient supplement (ThermoFisher A1517001) or DMEM/F-12 basal media (ThermoFisher 11320033) supplemented with insulin-transferring-selenium (2x) (ThermoFisher 41400045), sodium bicarbonate (1.74 g/L) (ThermoFisher 25080094), L-ascorbic acid 2-phosphate, hFGF2 (25 ng/ml) and TGFβ (2 ng/ml). The cells were incubated at 37 C and 5% CO_2 with media changed

daily. Typically, cells were maintained for up to one week before passaging. To passage the cells, DPBS was used to wash each well being passaged, followed by incubation at 37 °C with 0.5 mM EDTA in DPBS for 4 mins. The 0.5mM EDTA in DPBS was aspirated, fresh E8 media was added, and a pipette was used to flush the wells to detach the cells and break them into 5-10 cell clumps. The cells were transferred into a tube and allowed to settle, thus eliminating the small clumps and single cells. The supernatant was aspirated, the pellet was resuspended in complete E8 media and plated, typically in a 1:12 ratio split.

Lentivirus production

HEK 293T cells were expanded and subsequently passaged by washing with DPBS and incubating with 0.5% EDTA in DPBS. The cells were pelleted, resuspended in MEF media, counted, and plated in T150 flasks at a density of 1.8*107 cells per flask. The cells were allowed to attach for 24 hr at 37 °C before transfection with the three lentiviral (LV) plasmids. In 15ml Falcon tubes (one per virus being produced), 25 ug of pWPT-transgene vector cloned from the pWPT-GFP vector (addgene #12255), 14 ug of psPAX2 LV helper plasmid (addgene #12260), and 6 ug of pMD2.G LV packaging plasmid (addgene #12259) were added to room temperature basal RPMI media. The solution was vortexed, 68 ul of TurboFect transfection reagent (ThermoFisher R0532) was added, and complexes were formed for 30 min at room temperature. The complexes were gently pipetted into the T150 flasks containing the HEK cells, one flask per LV transfection, and incubated for 18-24 hr at 37 °C. Following the 18-24 hr transfection period, the cells were washed with DPBS and 15 ml of MEF media was added. After 24-36 hr, the supernatant was harvested and filtered through 0.45 µm filters. DNaseI (5 U/ml) and MgCl2 (1 mM) was added (optional due to the presence of Mg already in the supernatant media), and incubated at room temperature for 20 min. Clontech Lenti-X Concentration (Takara 631232) solution was used to concentration the LV batches by adding

5.0 ml of Lenti-X Concentrator to 15.0 ml of supernatant (1:3 ratio), and incubating at 4 °C for 30 min to 12 hr. The solution was centrifuged at 1500 x g, 4 °C for 45 min, after which the supernatant was aspirated, and the pellet was resuspended in 250 ul (1:100 of the pre concentrated LV containing supernatant volume). The LV batches were frozen at -80 °C in single-use aliquots for experiments to prevent drastic decreases in efficacy caused by free-thaw cycles.

Quantitative real-time PCR analyses.

Cellular RNA was extracted using the Sigma Aldrich GenElute Total RNA Purification Kit (RNB100-100RXN) following the manufacturer's suggested protocol, including the optional DNAse incubation step to increase RNA purity. The cDNA was produced using previously established reverse transcription methods. RNA quantity and purity was analysed, and 500 ng of RNA was added to each 11.8 ul reaction along with 0.5 ul of random primer (Promega C1181) and 1 ul of dNTP (Promega U1511). The remaining volume was brought up to 11.8 ul using nuclease-free water (ThermoFisher AM9937). The reactions were incubated at 65 °C for 5 mins to denature the RNA and random primers, then snap cooled on ice and placed at room temperature. A master mix of 4 ul of 5x 1st strand buffer (ThermoFisher 18064-071), 2 ul of 0.1M DTT, 0.5 ul RNase OUT (ThermoFisher 10777-019), and 0.2 ul of Superscript II (ThermoFisher 18064-071) was prepared per reaction, and scaled based on the number of reactions in the experiment. The master mix was vortexed and 6.7 ul of master mix was added to each reaction. The reactions were then incubated on a heat block at 25 °C for 10 mins, 42 °C for 50 mins, and lastly 70 °C for 15 mins. The final cDNA was diluted to 600 ul using nuclease-free water.

Reverse transcription was followed by qPCR in a 384-well format using biological triplicates and technical duplicates in each experiment. All primer pairs are diluted to 100 uM

stocks and subsequently diluted to 5 uM for use in qPCR. The reaction volumes in a 384-well non-skirted plate (ThermoFisher AB-0600) was 10 ul consisting of 0.4 of ul forward primer (Sigma Aldrich), 0.4 ul of reverse primer (Sigma Aldrich), 5.0 ul of Kapa Syber Fast Low Rox (Sigma Aldrich KK4622), 1.2 ul of nuclease-free water, and 3 ul of cDNA. The plates were sealed with optically clear adhesion covers (ThermoFisher 4360954) and run using the following cycling parameters: 95 °C for 3 mins, 95 °C for 5 secs, 60 °C for 30 secs, cycle to step 2 another 39 times, and lastly melt curve from 65 °C to 95 °C. All expression values ($2^{-\Delta Ct}$) were normalized to housekeeping genes PBGD and RPLP0.

Reads mapping

The 10X single cell sequencing data were mapped with the Cellranger program (version 2.0.2)[14] to the reference `refdata-cellranger-GRCh38-1.2.0', which is the human genome `GRCh38` downloaded from (https://support.10xgenomics.com/single-cell-gene-expression /software/downloads/latest). Reads for each sample were first imported into an `AnnData` object (https://anndata.readthedocs.io/en/latest/ index.html) in the SCANPY[19] framework, count matrices were then concatenated into a total matrix of 273,033 cells and 33,694 genes.

Normalisation and regression

Read counts were log-normalised in the SCANPY framework using total count normalisation (scaling factor 10,000). Highly variable genes were selected for downstream analysis based on their normalised dispersion, obtained by scaling with the mean and standard deviation of the dispersions for genes falling into a given bin for mean expression of genes (`highly_variable_genes` routine in SCANPY). Technical variation was regressed out using a linear regression model (`regress_out` function from the python package of `NaiveDE`[15]), which allows the user to specify wanted variance when cell types or stages are known.

Regressed values were scaled by maintaining the default maximum value of 10 for each gene expression in each cell (`scale` routine in SCANPY).

Clustering, annotation and dimensionality reduction

Clusters were detected by applying the graph-based, community detection method `Louvain`. Annotation of clusters was based on the expression of known markers. Since Louvain clustering does not necessarily correlate with biologically meaningful clusters and can over-cluster cells associated with the same cell type, clusters with the same annotation were merged. Principal Component Analysis (PCA), t-Distributed Stochastic Neighbor Embedding (TSNE)[20] and Uniform Manifold Approximation and Projection (UMAP)[18] plots were calculated with the SCANPY routines.

Graph-abstraction

Transcriptional similarity among stages was quantified and visualised using the method: Partition-Based Graph Abstraction (PAGA)[28]. PAGA generates a graph-like map of cells based on estimated connectivities which preserves both the continuous and disconnected structure of the data at multiple resolutions. In a PAGA graph, nodes correspond to cell groups and edge weights quantify the connectivity between groups. Such weights represent the confidence in the presence of an actual connection by allowing discarding spurious, noise-related connections. In our PAGA graphs, connectivity estimates were used to analyse the relative similarity among stages.

Analysis of published RNA-sequencing data

Bulk RNA-Seq counts for differentiated biliary organoids (DBO), fetal hepatocyte organoids (FHO), primary adult hepatocytes (PAH) and primary fetal hepatocytes (PFH) were

downloaded from GEO (series GSE111301, samples GSM3490461, GSM3490463, GSM3490466, GSM3490467, GSM3490468, GSM3490470). Similarly to *Hu et. al. 2018*, counts were normalised using the DESeq2 package in the R environment by applying the variance stabilising transformation. Cell Ranger feature-barcode matrices derived from hepatoblast organoids (HBO) were filtered, based on number of genes detected and percentage of reads that map to the mitochondrial genome (<0.1), log-normalised and averaged using the Seurat package. PCA analysis of the merged bulk and averaged single cell samples was performed using the prcomp package in R.
